# VAMP3/Syb and YKT6 are required for the fusion of constitutive secretory carriers with the plasma membrane

**DOI:** 10.1371/journal.pgen.1006698

**Published:** 2017-04-12

**Authors:** David E. Gordon, Joanne Chia, Kamburpola Jayawardena, Robin Antrobus, Frederic Bard, Andrew A. Peden

**Affiliations:** 1 University of California San Francisco, Department of Cellular and Molecular Pharmacology, San Francisco, CA, United States of America; 2 Institute of Molecular and Cell Biology, 61 Biopolis Drive, Proteos, Singapore; 3 Cambridge Institute for Medical Research, University of Cambridge, Hills Road, Cambridge, United Kingdom; 4 Department of Biomedical Science & Centre for Membrane Interactions and Dynamics (CMIAD), The University of Sheffield, Western Bank, Sheffield, United Kingdom; The University of North Carolina at Chapel Hill, UNITED STATES

## Abstract

The cellular machinery required for the fusion of constitutive secretory vesicles with the plasma membrane in metazoans remains poorly defined. To address this problem we have developed a powerful, quantitative assay for measuring secretion and used it in combination with combinatorial gene depletion studies in *Drosophila* cells. This has allowed us to identify at least three SNARE complexes mediating Golgi to PM transport (STX1, SNAP24/29 and Syb; STX1, SNAP24/29 and YKT6; STX4, SNAP24 and Syb). RNAi mediated depletion of YKT6 and VAMP3 in mammalian cells also blocks constitutive secretion suggesting that YKT6 has an evolutionarily conserved role in this process. The unexpected role of YKT6 in plasma membrane fusion may in part explain why RNAi and gene disruption studies have failed to produce the expected phenotypes in higher eukaryotes.

## Author summary

The constitutive secretory pathway delivers newly synthesised proteins and lipids to the cell surface and is essential for cell growth and viability. This pathway is required for the secretion of molecules such as antibodies, cytokines and extracellular matrix components so has both significant physiological and commercial importance. The majority of secreted proteins begin their journey at the endoplasmic reticulum, pass through the Golgi, and are transported to the cell surface in small vesicles/tubules which fuse with the plasma membrane. Surprisingly, the molecular understanding of this fusion step is still unclear and in higher eukaryotes it is not known which SNARE proteins drive this process. To address this problem we have developed a powerful, quantitative assay for measuring secretion and used it in combination with gene depletion studies in *Drosophila* cells. Using this assay we identified three SNARE complexes driving the fusion of secretory vesicles with the plasma membrane and uncovered an unexpected role for the R-SNARE YKT6 in this process. Using this knowledge we have re-examined the role of SNAREs in the fusion of secretory carriers with the plasma membrane in mammalian cells and have found that YKT6 has an evolutionarily conserved role in this process.

## Introduction

Constitutive secretion delivers newly synthesised proteins and lipids to the cell surface and is essential for cell growth and viability. This pathway is required for the exocytosis of molecules such as antibodies, cytokines and extracellular matrix components so has both significant physiological and commercial importance. The majority of constitutive secreted proteins are synthesised at the endoplasmic reticulum, pass through the Golgi, and are transported to the cell surface in small vesicles and tubules which fuse with the plasma membrane [1, 2]. Constitutive secretory vesicles are not stored within the cell and do not require a signal to trigger their fusion with the plasma membrane which is in contrast to dense core secretory granules or synaptic vesicles [3, 4]. In some cell types, such as MDCK cells and macrophages, there is evidence that constitutive secretory cargo passes through a endosomal intermediate on its way to the cell surface [5, 6]. However, in non-polarised cells endosomal intermediates do not appear to play a major role in this pathway [7].

Vesicle fusion is driven by a family of molecules known as SNAREs. SNARE are generally small (14-42kDa), C-terminally anchored proteins that have a highly conserved region termed the SNARE motif that has the ability to interact with other SNAREs [8, 9]. For membrane fusion to occur, SNAREs on opposing membranes must come together and their SNARE motifs zipper up to form a SNARE complex [10, 11]. Detailed characterisation of the neuronal SNARE complex (syntaxin 1A/VAMP2/SNAP25) required for synaptic vesicle fusion has provided a mechanistic framework for understanding the function of SNAREs [4, 12, 13]. There are 38 SNAREs encoded in the human genome and they can be classified as either R or Q-SNAREs depending on the presence of a conserved arginine or glutamine in their SNARE motif [14–16]. Q-SNAREs can be further subdivided into Q_a_, Q_b_ and Q_c_ SNAREs based on their homology to syntaxin and SNAP25. A typical fusogenic SNARE complex will contain four SNARE motifs (Q_a_, Q_b_, Q_c_ and R)[17]. Q_bc_-SNAREs such as SNAP23, 25, 29 and 47 contribute two SNARE motifs to the SNARE complex. R-SNAREs can also be further classified as either longin or brevin type SNAREs. Longin type R-SNAREs contain a longin type fold and are found in all eukaryotes and while brevin type SNAREs are less widely conserved across species [18].

Over the past twenty years significant progress has been made defining the SNARE complexes required for the majority of intracellular transport steps within eukaryotic cells (reviewed in [19–23]). In addition, there are an increasing number of examples where the SNARE complexes required for the secretion of specific cargo such as Wnt, TNF and IL-6 have been identified [24–26]. However, these proteins are not delivered directly to the cell surface from the TGN but pass through an endosomal compartment. Many labs, including our own, have attempted to identify the machinery which drive the fusion of constitutive secretory vesicles with the plasma membrane and on the whole very little progress has been made [27–34]. This in part may be due to the fact that there are multiple routes to the cell surface from the Golgi and redundancy in the fusion machinery. If we just consider the R-SNAREs, the human genome encodes seven post-Golgi SNAREs (Table 1) and a typical mammalian cell line can express at least five R-SNAREs so disruption of just one R-SNARE is unlikely to block secretion if they are functionally redundant [15, 27]. To overcome this problem we have decided to analyse SNARE function in *Drosophila* cells as they have a simpler genome with less redundancy. The *Drosophila* genome encodes 26 SNAREs with 16 of them predicted to be localised to post-Golgi membranes based on their homology to mammalian SNAREs [14, 15]. The complexity is reduced even further as *Drosophila* cell lines just express two post-Golgi R-SNAREs, Syb and VAMP7 (based on publically available microarray data generated by the modENCODE project)[35].

**Table 1:** R-SNARE homologues in different organisms.

*S*. *cerevisiae*	*D*. *melanogaster*	*H*. *sapiens*
SEC22	Sec22b*	Sec22b*
YKT6	YKT6*	YKT6*
SNC1 / SNC2	nSyb / Syb*	VAMP1/ VAMP2*/ VAMP3*/ VAMP4*/ VAMP5/ VAMP8*
NYV1	VAMP7*	VAMP7*

The number of post-Golgi R-SNAREs has significantly increased in higher eukaryotes. A detailed phylogenetic analysis of SNARE sequences can be found in [15]. The SNARE database website (http://bioinformatics.mpibpc.mpg.de/snare/index.jsp) was used to help generate this table.

* Indicates the SNARE is expressed in the reporter cell lines (C1 and C3) used in this study. For simplicity we have included VAMP4 and VAMP8 in the ‘brevin’ group.

In this study, we have developed a novel, quantitative assay for measuring constitutive secretion based on a reporter cell line that can be effectively used to monitor secretion by flow cytometry, immunoblotting and fluorescence microscopy. Depletion of known components of the secretory pathway in *Drosophila* cells (STX5, SLH and ROP) causes robust blocks in ER to Golgi and Golgi to plasma membrane transport, therefore validating this approach. As predicted, there is redundancy in the post-Golgi SNAREs and multiple SNAREs must be depleted to obtain robust blocks in secretion. We have detected strong negative genetic interactions between *Drosophila* STX1 and STX4, SNAP24 and SNAP29, STX1 and Syb, and SNAP24 and Syb. We have also detected a novel and unexpected genetic interaction between Syb and YKT6. Depletion of YKT6 and VAMP3 in mammalian cells also causes a robust block in secretion indicating that this negative genetic interaction is conserved across species and provides evidence that these two R-SNAREs function in the late secretory pathway.

## Results

### Development of a novel *Drosophila* cell line (C3) for measuring constitutive secretion

**Fig 1:**
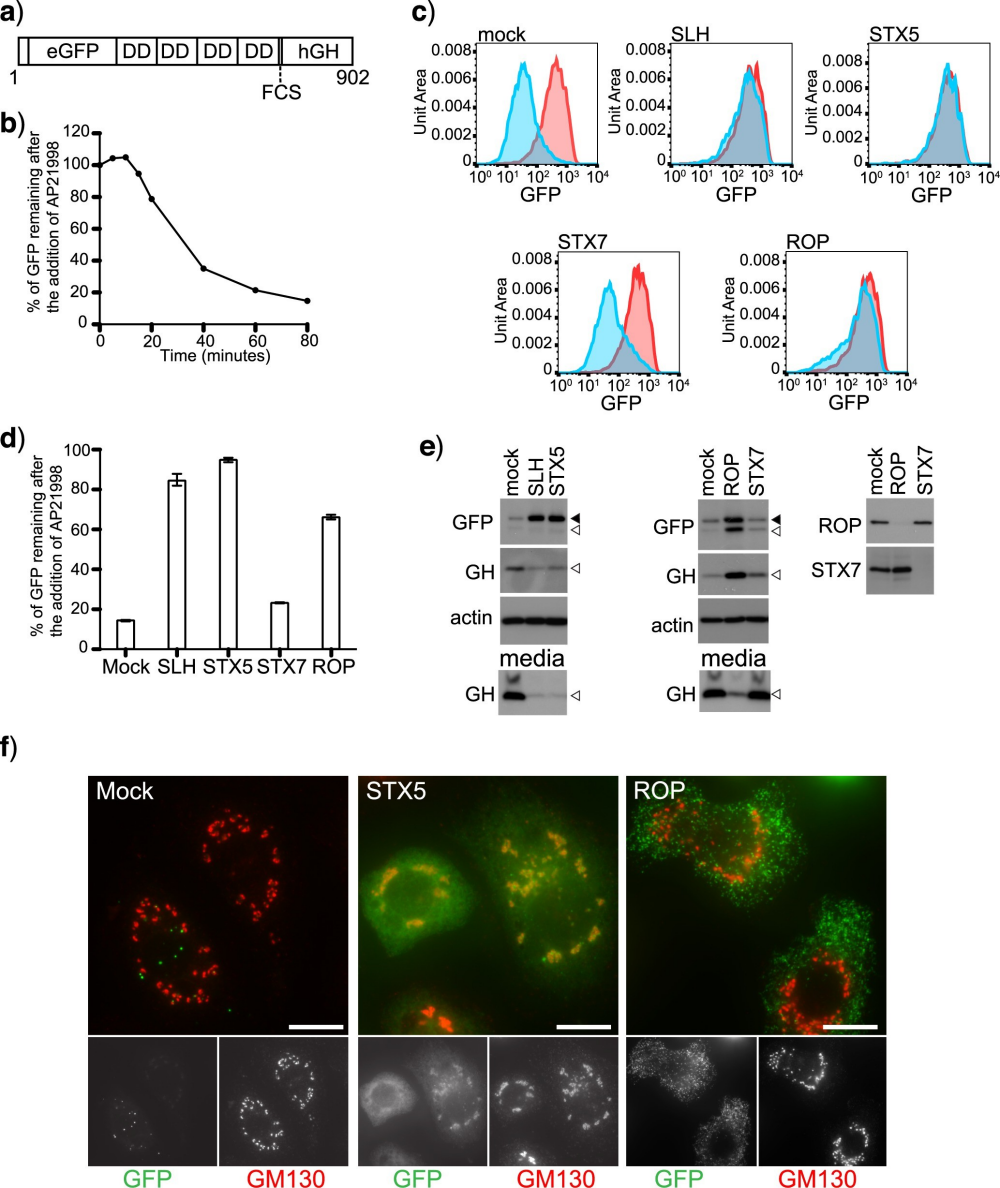
Development of a novel assay for measuring secretion in *Drosophila* cells. A) Schematic of the reporter construct used to measure secretion (DD, dimerisation domain; FCS, furin cleave site; hGH, human growth hormone and numbers indicate amino acids). B) Transport kinetics of the reporter construct were determined by incubating C3 cells with AP21998 at 25°C for the indicated times and the mean fluorescence of the cells measured using flow cytometry. The amount of cargo remaining in the cells after the addition of AP21998 was calculated as a ratio between the control sample (no AP21998) and the experimental samples (+AP21998). C) Clone 3 cells were mock transfected (TransFast only) or transfected with dsRNA targeting the indicated genes. After 96 hours, the cells were incubated with AP21998 at 25°C for 80 minutes and their mean fluorescence determined using flow cytometry. The red histogram indicates the fluorescent intensity of the control sample, no AP21998 and the blue histogram shows the fluorescent intensity of the cells incubated with AP21998. D) The amount of cargo remaining in the cells was calculated as in B and plotted (Error bars show experimental range for three repeats). E) Clone 3 cells were mock transfected (TransFast only) or transfected with dsRNA targeting the indicated genes. After 96 hours, the cells were incubated with AP21998 at 25°C for 80 minutes and the media and cells harvested for immunoblotting. Solid arrowhead indicates unprocessed cargo and unfilled arrowhead furin processed cargo. F) Clone 3 cells were either mock transfected or transfected with dsRNA targeting STX5 and ROP. After 72 hours the cells were seeded onto coverslips. The next day, the cells were incubated with AP21998 at 25°C for 80 minutes. The cells were then fixed and stained for the Golgi marker GM130. Scale Bar 10μm.

We previously used a ligand-inducible reporter system to measure constitutive secretion in mammalian cells [27, 36]. This system utilizes a GFP-tagged reporter construct (cargo) that is retained in the ER until the addition of a small molecule (AP21998 or D/D solubiliser), which causes the cargo to exit the ER in a synchronous pulse (Fig 1A). The transport of the cargo can be monitored using flow cytometry, microscopy and immunoblotting. The cargo contains a furin cleavage site so changes in its molecular weight can be used to determine if it has reached the trans-Golgi network (TGN), where the furin endoprotease normally resides. We have moved this reporter system into *Drosophila* S2 cells and generated a clonal cell line (C3). C3 cells have similar secretion kinetics to mammalian cells and secrete approximately 80% of their cargo in 80 minutes (Fig 1B) [27].

To validate the C3 cells we used RNAi to deplete the *Drosophila* orthologues of syntaxin 5 (STX5) and Sly1 (SLH), genes previously shown to be essential for ER-Golgi transport in human cells [27, 37, 38]. Amplicons to both of these genes were designed using FLYBASE, synthesised and transfected in to C3 cells. The mRNA level for both STX5 and SLH were reduced by over 80% as determined by qRT-PCR (S1 Fig). Depletion of STX5 and SLH cause a significant block in biosynthetic transport as almost no cargo is secreted from the cells as determined by flow cytometry (Fig 1C and 1D) and immunoblotting (Fig 1E). Similar results were obtained using alternate amplicons indicating that the observed block in secretion is not due to off-target effects (S1 Table). In the STX5 depleted cells the trapped cargo is found in the Golgi (co-localisation with Golgi marker GM130) and reticular and tubular structures most likely the ER (Fig 1F and S1 Fig).

To determine whether the assay could be used to detect blocks in post-Golgi trafficking we depleted ROP, the *Drosophila* Sec1 homolog [39, 40] and STX7/Avalanche an endosomal Q-SNARE. Immunoblotting for ROP and STX7 confirmed that both proteins were efficiently depleted (Fig 1E). Depletion of ROP caused a significant defect in secretion, while depletion of the endocytic SNARE STX7 did not (Fig 1C–1E). An alternate ROP amplicon give a similar phenotype indicating that the defect in secretion is not due to off-target effects (S1 Table). In the ROP depleted cells, a significant proportion of the retained cargo has been furin-processed suggesting that it has reached a post-Golgi compartment (Fig 1E, appearance of lower molecular weight band in GFP blot and accumulation of processed GH in the cells). In support of the biochemical data we observe cargo accumulating in small vesicular structures in the ROP depleted cells (Fig 1F). These membranes are distinct from the Golgi (GM130 negative) and start appearing approximately 20 minutes after the induction of secretion suggesting that they may be post-Golgi transport carriers which have been unable to fuse with the plasma membrane.

### STX1, STX4 and Syb are required for the fusion of secretory carriers with the plasma membrane

**Fig 2:**
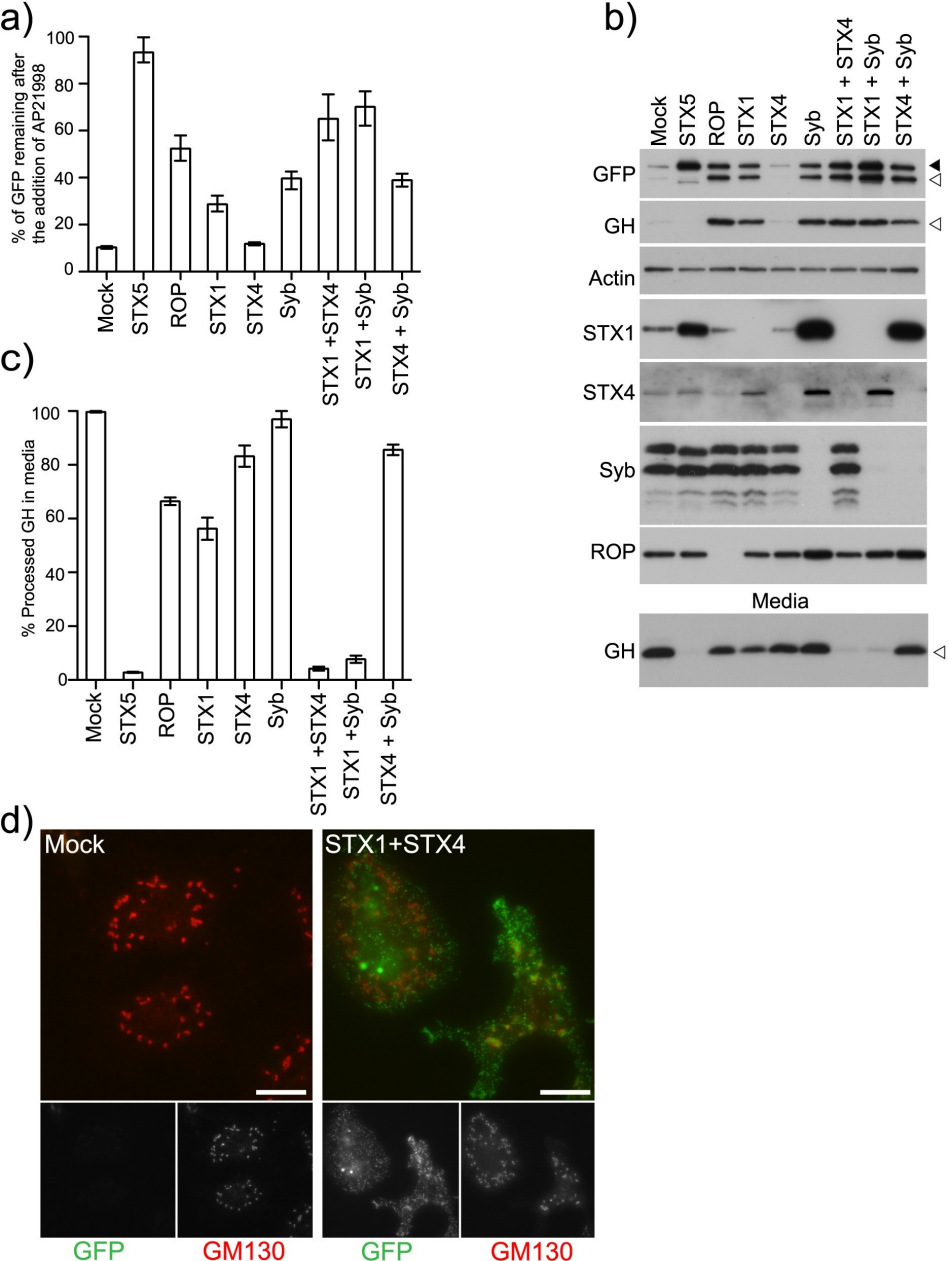
STX1, STX4 and Syb are required for the fusion of secretory carriers with the plasma membrane. A) Clone 3 cells were mock transfected (TransFast only) or transfected with dsRNA targeting the indicated genes. After 96 hours, the cells were incubated with AP21998 at 25°C for 80 minutes and their mean fluorescence determined using flow cytometry. The amount of cargo remaining in the cells was calculated and plotted (Error bars show experimental range for six repeats). B) Clone 3 cells were mock transfected (TransFast only) or transfected with dsRNA targeting the indicated genes. After 96 hours, the cells were incubated with AP21998 at 25°C for 80 minutes and the media and cells harvested for immunoblotting. Solid arrowhead indicates unprocessed cargo and unfilled arrowhead furin processed cargo. The amount of processed GH in the media was quantified using densitometry and plotted in C (Error bars show experimental range for two repeats). *The apparent increase in STX1 and STX4 levels by immunoblotting when STX5 and Syb are depleted is not because of a change in total STX1 levels but is due to a change in its extractability from cells. No difference in STX1 or STX4 levels were observed when cells are directly prepared in Laemmli sample buffer (S2 Fig). D) Clone 3 cells were either mock transfected or transfected with dsRNA targeting STX1 and STX4. After 72 hours the cells were seeded onto coverslips. The next day, the cells were incubated with AP21998 at 25°C for 80 minutes. The cells were then fixed and stained for the Golgi marker GM130. Scale Bar 10μm.

To determine which post-Golgi SNAREs mediate fusion of biosynthetic vesicles with the plasma membrane we depleted syntaxin 1 (STX1), syntaxin 4 (STX4) and synaptobrevin (Syb). These SNAREs are the closest homologs of the yeast genes SSO1/2 and SNC1/2 previously shown to mediate the fusion of biosynthetic vesicles with the plasma membrane [41, 42]. We depleted these SNAREs individually, or in combination and the knock down efficiency was determined by immunoblotting and RT-PCR (Fig 2B and S2 Fig). Depletion of STX1 or Syb leads to a partial block in secretion while depletion of STX4 had no effect (Fig 2A and 2C)(S2 Fig). Depletion of STX1 or Syb leads to a similar phenotype to that observed with ROP knock down, where furin-processed cargo is retained inside the cell (Fig 2B). The block in secretion became more pronounced when STX1 and STX4, or STX1 and Syb were depleted in combination indicating a negative genetic interaction between these genes. In the STX1-STX4 depleted cells the retained cargo is found in small vesicular structures scattered throughout the cytoplasm (Fig 2D). No genetic interaction was detected between STX4 and Syb. The STX1-Syb genetic interaction can be replicated using an alternative amplicons targeting Syb. Alternative amplicons targeting STX1 did not efficiently knockdown the protein (S1 Table).

### YKT6 has a role in the fusion of secretory carriers with the plasma membrane

As depletion of Syb did not produce a complete block in secretion it was possible that another R-SNARE might be able to substitute for the loss of Syb. To address this we used publically available microarray data to determine which *Drosophila* R-SNAREs are expressed in S2 cells (modENCODE project). The R-SNAREs Syb, VAMP7, YKT6 and Sec22b are expressed in S2 cells, but not the neuronal R-SNARE n-Syb. This is consistent with previous studies indicating that n-Syb is exclusively expressed in neuronal tissue [43].

**Fig 3:**
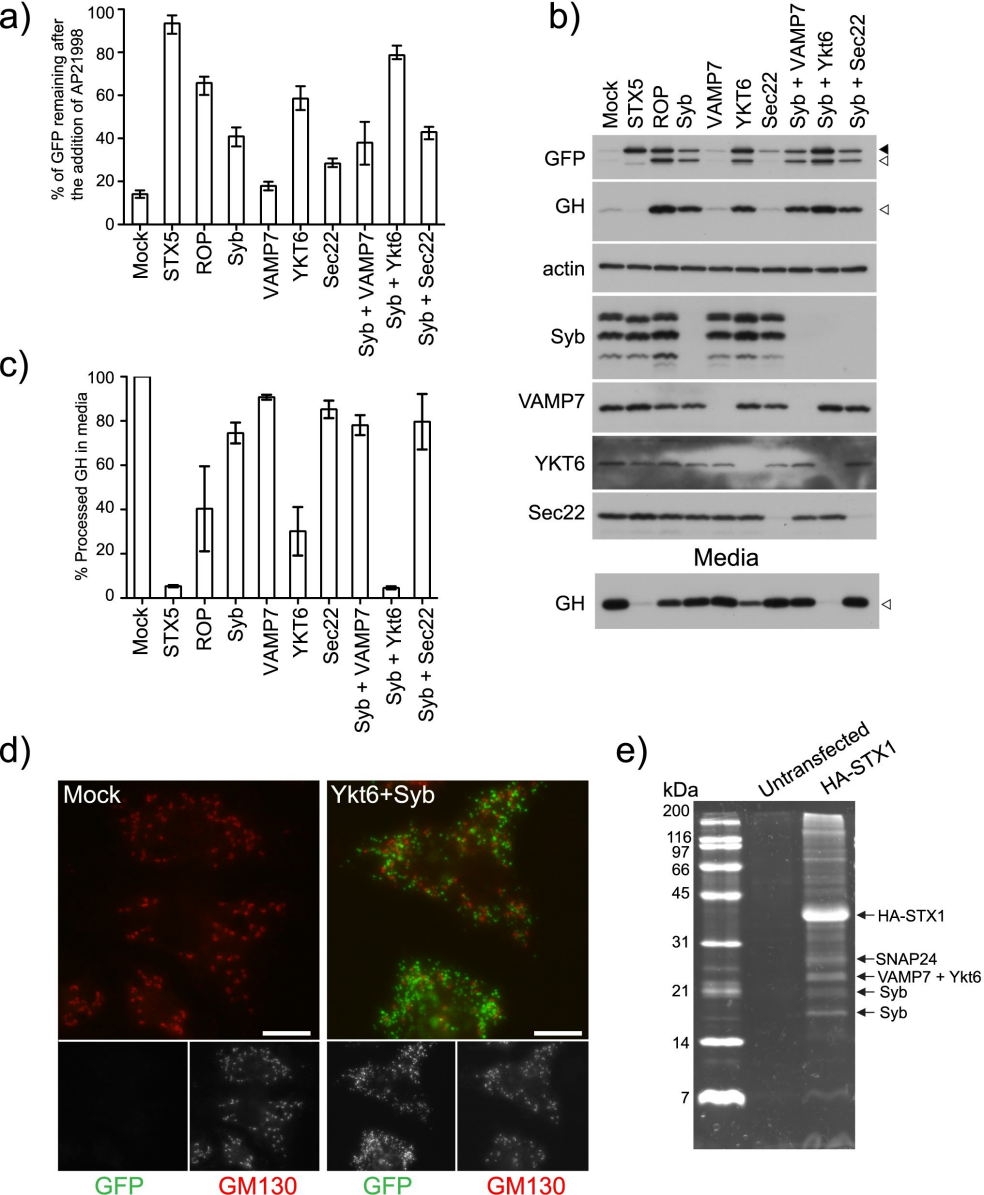
Syb and YKT6 have a role in the fusion of secretory carriers with the plasma membrane. A) Clone 3 cells were mock transfected (TransFast only) or transfected with dsRNA targeting the indicated genes. After 96 hours, the cells were incubated with AP21998 at 25°C for 80 minutes and their mean fluorescence determined using flow cytometry. The amount of cargo remaining in the cells was calculated and plotted (Error bars show experimental range for six repeats). B) Clone 3 cells were mock transfected (TransFast only) or transfected with dsRNA targeting the indicated genes. After 96 hours, the cells were incubated with AP21998 at 25°C for 80 minutes and the media and cells harvested for immunoblotting. Solid arrowhead indicates unprocessed cargo and unfilled arrowhead furin processed cargo. The amount of processed GH in the media was quantified using densitometry and plotted in C (Error bars show experimental range for two repeats). D) Clone 3 cells were either mock transfected or transfected with dsRNA targeting YKT6 and Syb. After 72 hours the cells were seeded onto coverslips. The next day, the cells were incubated with AP21998 at 25°C for 80 minutes. The cells were then fixed and stained for the Golgi marker GM130. Scale Bar 10μm. E) To determine which SNAREs interact with STX1 a native immunoprecipitation was performed from *Drosophila* cells stably expressing HA tagged STX1. The isolated protein complexes were separated by SDS-PAGE and the major bands identified using mass-spectrometry.

We depleted the R-SNAREs individually or in combination and determined the knock down efficiency by immunoblotting (Fig 3B). Depletion of Syb, YKT6 and Sec22b caused a partial block in secretion as determined by flow cytometry and immunoblotting of the cargo (Fig 3A–3C) (S3 Fig). Depletion of Syb or YKT6 causes retention of furin-processed cargo indicating a late block in secretion (Fig 3B, GFP and GH blots). This block became more severe when YKT6 and Syb were depleted in combination. The level of block was comparable to that observed when STX5 is depleted as almost no processed GH was detected in the media (Fig 3B, GH media blot). In support of the role of Syb and YKT6 in the fusion of secretory carriers with the plasma membrane we observe an accumulation of secretory carriers in cells depleted for both of these genes (Fig 3D). The observed genetic interaction between Syb and YKT6 are not due to off-target effects as they can be reproduced using alternate amplicons targeting both genes (S1 Table). Importantly, no genetic interaction was detected between the R-SNARE Sec22b and Syb indicating that YKT6-Syb interaction is specific and not due to general toxicity (Fig 3A–3C). In support of YKT6 having a role in the fusion of secretory carriers with the plasma membrane we were able to immuoprecipitate YKT6 in a complex with STX1 from S2 cells (Fig 3E) (Table 2).

**Table 2:** Proteins identified co-immunoprecipitating with STX1.

	Score (PMF/MSMS)	Unique peptides (coverage)
Syntaxin-1A	350/248	15 (48%)/4 (14%)
SNAP24	93/-	25 (81%)/-
VAMP7	116/-	20 (64%)/-
Ykt6	105/-	17 (72%)/-
Synaptobrevin	207/140	6 (66%)/2 (20%)
Synaptobrevin	226/142	5 (37%)/2 (20%)

Proteins were identified by MALDI-MS and MALDI-MSMS where successful. Identified proteins are shown with score obtained either by peptide mass fingerprinting (PMF) or MSMS with the number of unique peptides and corresponding sequence coverage shown in each case.

### YKT6 is required at multiple steps in the secretory pathway

**Fig 4:**
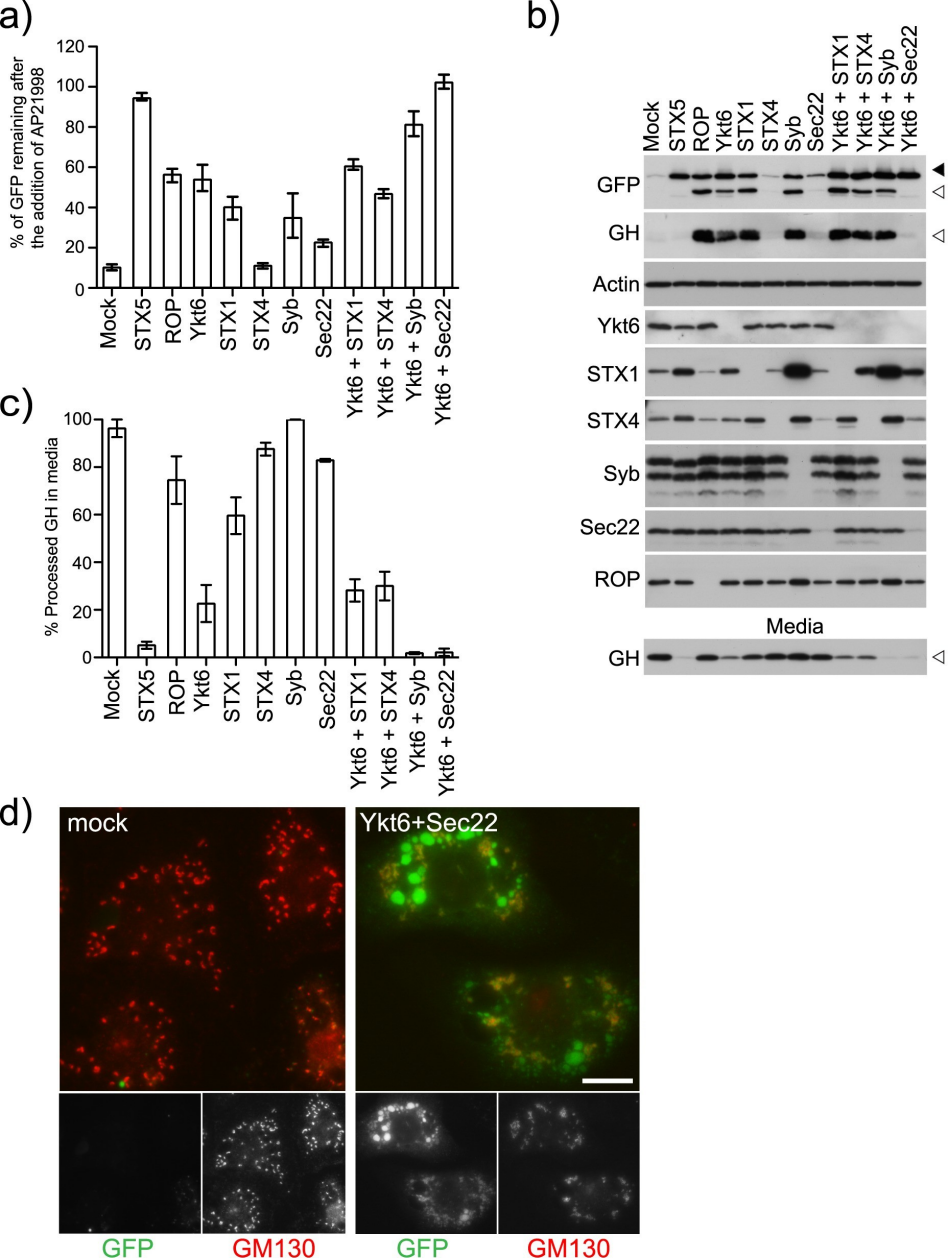
YKT6 functions on multiple intracellular pathways. A) Clone 3 cells were mock transfected (TransFast only) or transfected with dsRNA targeting the indicated genes. After 96 hours, the cells were incubated with AP21998 at 25°C for 80 minutes and their mean fluorescence determined using flow cytometry. The amount of cargo remaining in the cells was calculated and plotted (Error bars show experimental range for six repeats). B) Clone 3 cells were mock transfected (TransFast only) or transfected with dsRNA targeting the indicated genes. After 96 hours, the cells were incubated with AP21998 at 25°C for 80 minutes and the media and cells harvested for immunoblotting. Solid arrowhead indicates unprocessed cargo and unfilled arrowhead furin processed cargo. The amount of processed GH in the media was quantified using densitometry and plotted in C (Error bars show experimental range for two repeats). D) Clone 3 cells were either mock transfected or transfected with dsRNA targeting YKT6 and Sec22b. After 72 hours the cells were seeded onto coverslips. The next day, the cells were incubated with AP21998 at 25°C for 80 minutes. The cells were then fixed and stained for the Golgi marker GM130. Scale Bar 10μm.

In *S*. *cerevisiae*, it has previously been reported that YKT6 and Sec22 function redundantly in ER to Golgi transport [44]. To determine if this is also the case in *Drosophila* cells we depleted YKT6 and Sec22b individually and in combination (Fig 4B). As in *S*. *cerevisiae*, we see a robust block in constitutive secretion when YKT6 and Sec22b are depleted in combination (Fig 4A and 4C)(S4 Fig). The level of inhibition is very similar to that seen when STX5 is depleted. In the Sec22b/YKT6 depleted cells the cargo is trapped in the ER and has failed to reach the Golgi (Fig 4B and 4D). This is in contrast to what is observed when YKT6/Syb are depleted where there is an accumulation of furin processed cargo (Fig 4B). We also depleted YKT6 in combination with STX1 and STX4. No genetic interaction was detected between YKT6 and STX1 or YKT6 and STX4 (Fig 4A and 4C).

### SNAP24 and SNAP29 mediate the fusion of secretory carriers with the plasma membrane

**Fig 5:**
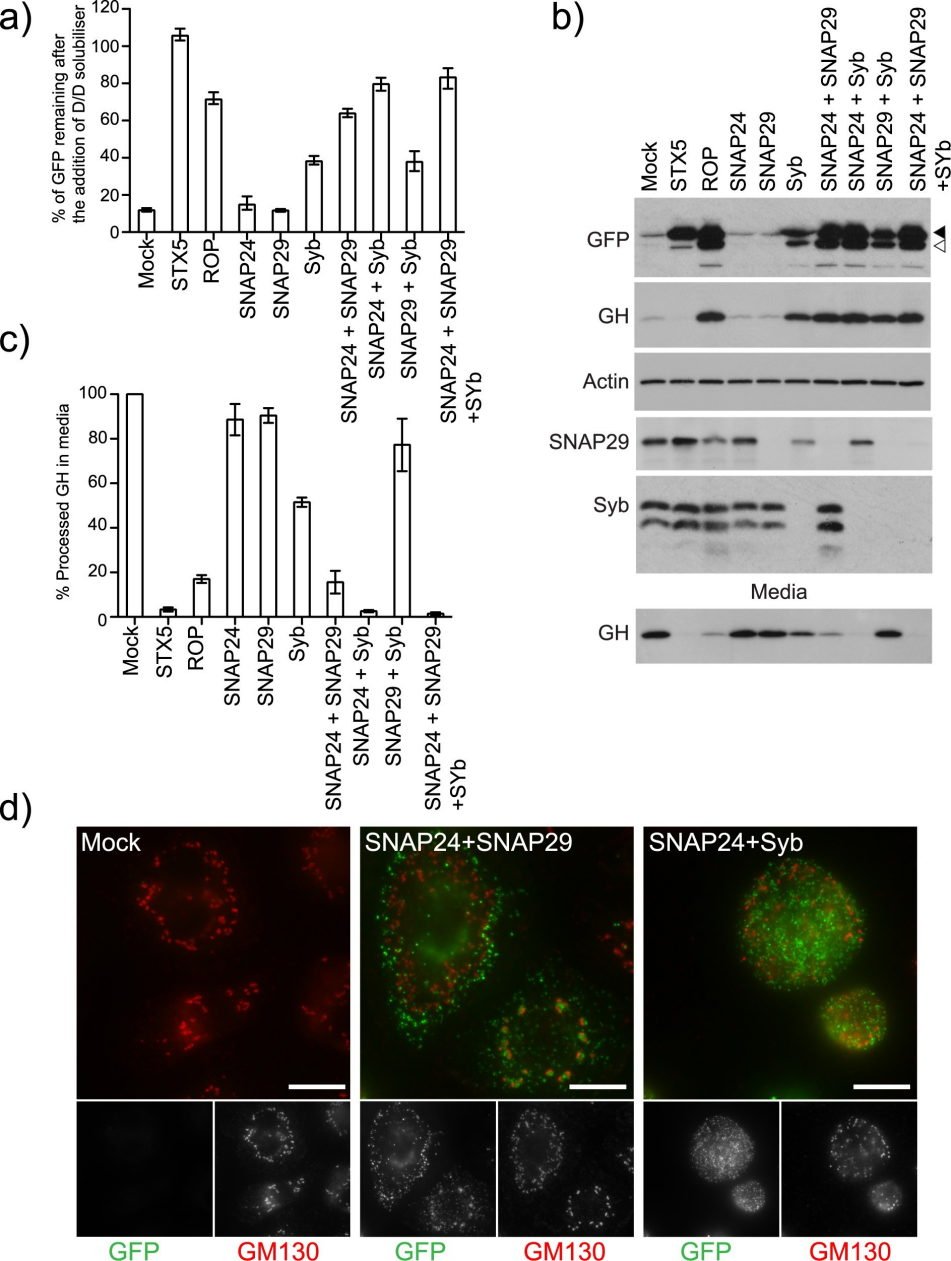
SNAP24 and SNAP29 mediate the fusion of secretory carriers with the plasma membrane. A) Clone 3 cells were mock transfected (TransFast only) or transfected with dsRNA targeting the indicated genes. After 96 hours, the cells were incubated with DD solubiliser at 25°C for 80 minutes and their mean fluorescence determined using flow cytometry. The amount of cargo remaining in the cells was calculated and plotted (Error bars show experimental range for three repeats). B) Clone 3 cells were mock transfected (TransFast only) or transfected with dsRNA targeting the indicated genes. After 96 hours, the cells were incubated with DD solubiliser at 25°C for 80 minutes and the media and cells harvested for immunoblotting. Solid arrowhead indicates unprocessed cargo and unfilled arrowhead furin processed cargo. The amount of processed GH in the media was quantified using densitometry and plotted in C (Error bars show experimental range for two repeats). D) Clone 3 cells were either mock transfected or transfected with dsRNA targeting the indicated genes. After 72 hours the cells were seeded onto coverslips. The next day, the cells were incubated with AP21998 at 25°C for 80 minutes. The cells were then fixed and stained for the Golgi marker GM130. Scale Bar 10μm.

Our data suggests that the Qa-SNAREs STX1/4 and the R-SNAREs Syb and YKT6 mediate the fusion of secretory carriers with the plasma membrane. A canonical SNARE complex also requires Qb and Qc SNARE domains, often provided by a Qbc-SNARE. The *Drosophila* genome encodes three SNAP genes: SNAP24, 25 and 29 (ubisnap) [15]. Only SNAP24 and SNAP29 are expressed in S2 cells based on publically available microarray data (modENCODE project). We depleted SNAP24 and SNAP29 individually or in combination and validated the knock down for SNAP29 using immunoblotting (Fig 5B). Depletion of SNAP24 or SNAP29 did not block secretion of the reporter construct. However, depletion of SNAP24 and SNAP29 in combination caused a significant block in secretion (Fig 5A–5C) (S5 Fig), similar to that seen when ROP is depleted. Similar results were obtained using alternate amplicons indicating that the observed block in secretion is not due to off-target effects (S1 Table). In the SNAP24-SNAP29 depleted cells a significant amount of furin-processed cargo is retained within the cells suggesting a late block in secretion (Fig 5B, GH and GFP blots). In support of the biochemical data we observe an accumulation of secretory carriers is the cells depleted for both SNAP24 and SNAP29 (Fig 5D). Consistent with SNAP24 having a role in the fusion of secretory vesicles with the plasma membrane we were able to immunoprecipitate SNAP24 in a complex with STX1 (Fig 3E)(Table 2).

We also investigated the effect of depleting SNAP24 and SNAP29 in combination with Syb. Depletion of SNAP24 and Syb in combination caused a robust block in secretion (Fig 5A–5C). The retained cargo was furin-processed indicating a late block in secretion (Fig 5B, GH and GFP blots). As in the SNAP24-SNAP29 knock down the cargo accumulated in small transport vesicles which did not co-localise with the Golgi (Fig 5D). No genetic interaction was detected between Syb and SNAP29. Depletion of SNAP29 in combination with SNAP24 and Syb did not cause a stronger block in secretion. In support of these observations we obtained similar results using alternate amplicons (S1 Table).

### Genetic interactions support a role for YKT6 in the mammalian late secretory pathway

**Fig 6:**
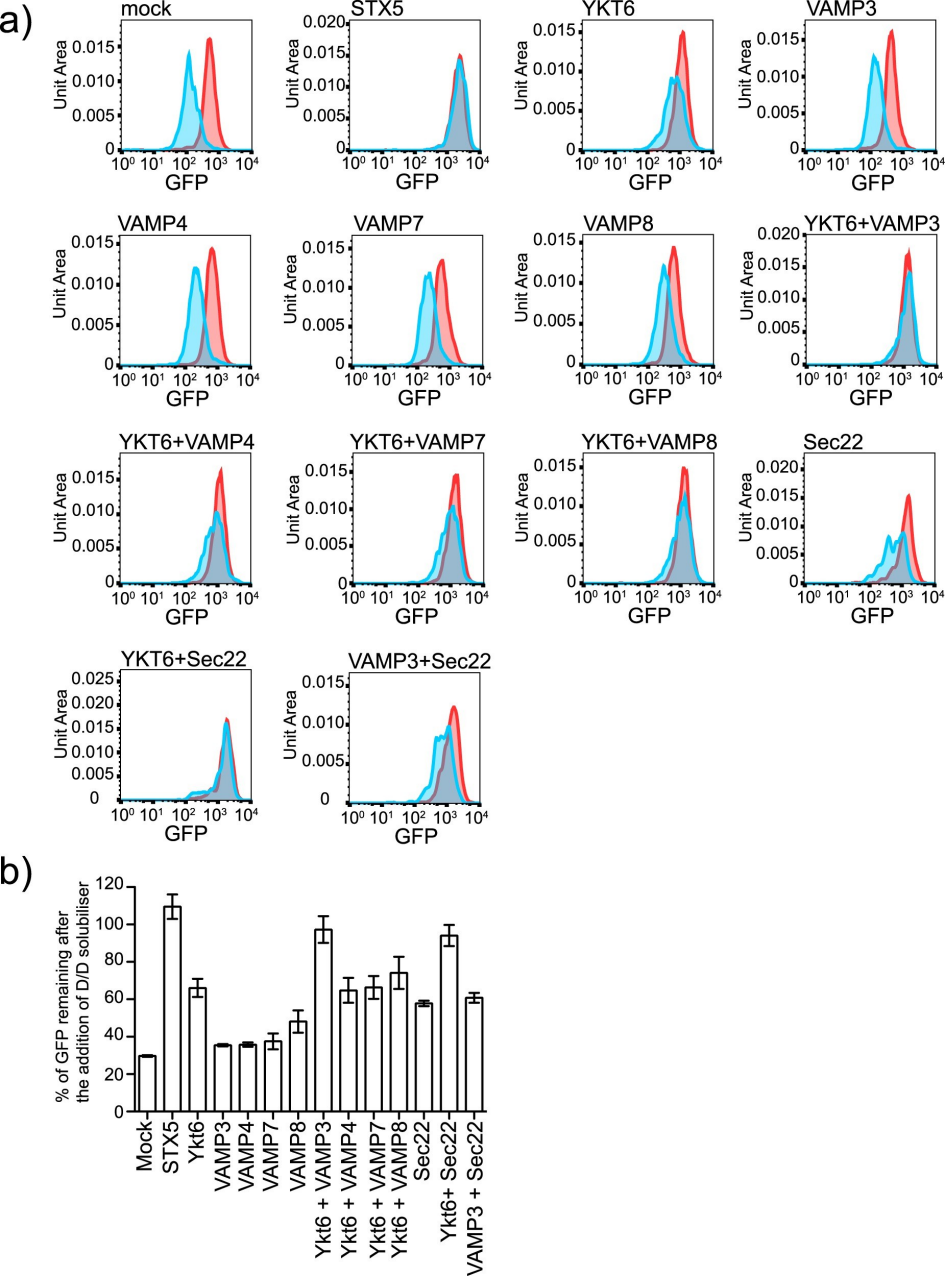
Genetic interactions support a role for YKT6 in the mammalian late secretory pathway. A) Representative histograms from a secretion experiment. Clone 1 cells were mock transfected (Oligofectamine only) or transfected with siRNA targeting the indicated genes. After 96 hours, the cells were incubated with D/D solubiliser at 37°C for 80 minutes and their mean fluorescence determined using flow cytometry. The red histogram shows the fluorescent intensity of the control sample, no DD solubiliser and the blue histogram shows the fluorescent intensity of the cells incubated with DD solubiliser. The amount of cargo remaining in the cells was calculated and plotted in B (Error bars show experimental standard deviation for three repeats).

We have uncovered an unexpected role for YKT6 in the fusion of biosynthetic vesicles with the plasma membrane in *Drosophila* cells. We next sought to determine if human YKT6 has a similar role. We have previously shown that combinatorial depletion of the human post-Golgi R-SNAREs VAMP3, 4, 7, and 8 does not block secretion in HeLa cells [27]. We depleted VAMPs 3, 4, 7, 8, and YKT6 individually or combination and determined the effect on secretion using our mammalian reporter line (HeLa-M C1). As previously reported depletion of VAMPs 3, 4, 7 and 8 individually causes little retention of the secretory cargo (Fig 6A and 6B). However, depletion of YKT6 causes partial retention of the cargo consistent with our previous results [27]. As in *Drosophila* cells combinatorial depletion of YKT6 and VAMP3 causes an almost complete block in secretion. This genetic interaction is specific because no interaction was detected with either VAMP4 or VAMP7. To investigate the specificity of the genetic interaction further we depleted the R-SNARE Sec22b in combination with YKT6 or VAMP3 (Fig 6A and 6B). As observed in *Drosophila* cells (Fig 3A–3C) no genetic interaction was detected between Sec22b and VAMP3 suggesting that the observed phenotype when Syb and YKT6 are depleted is not simply caused by a general defect in trafficking or toxicity. As in *S*. *cerevisiae* and the *Drosophila* cells we detect a strong negative genetic interaction between Sec22b and YKT6 [44].

## Discussion

**Fig 7:**
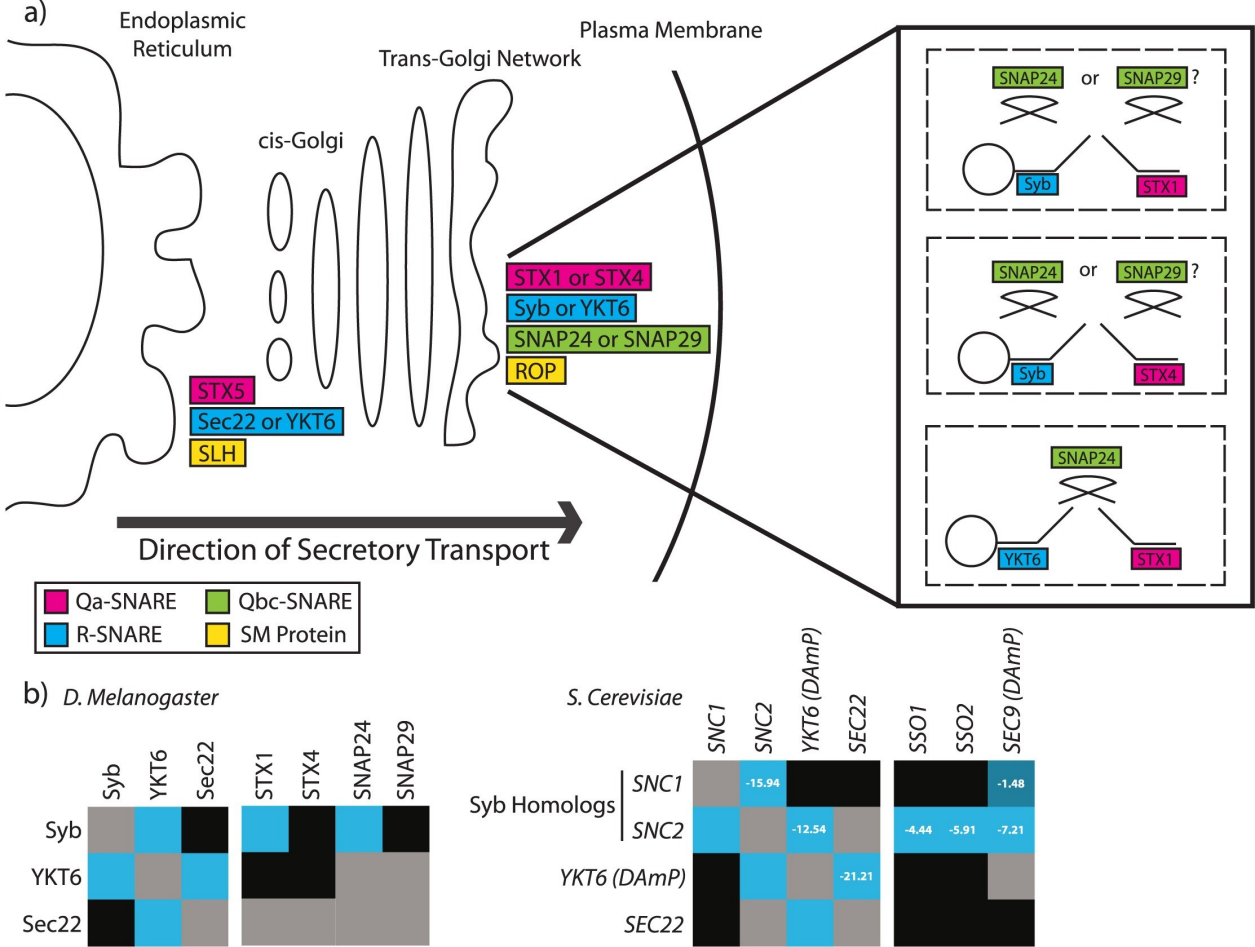
At least three SNARE complexes function in the fusion of secretory vesicles with the *Drosophila* plasma membrane A) Diagram summarising the functional and biochemical data presented in this study. Our data supports the role of at least three SNARE complexes acting at the plasma membrane in *Drosophila* cells. B) Diagram summarising the *Drosophila* genetic interactions in relation to published *S*. *cerevisiae* interaction data [58]. Figure reprinted with permission of the authors. S-Scores were calculated based on colony growth of double mutants as previously described in [63]. Blue indicates negative genetic interactions, black indicates no/neutral genetic interactions |S-score| < 0.5, and grey indicates no data for this gene combination (ND). To characterize essential genes in yeast, a strategy known as decreased abundance by mRNA perturbation (DAmP) was used to generate hypomorphic strains (analogous to gene knockdown). A |S-score| > 3 generally indicates a high confidence genetic interaction.

The aim of this study was to identify the SNAREs required for the fusion of constitutive secretory carriers with the plasma membrane in higher eukaryotes. To address this we have developed a simple and robust assay for measuring secretion in *Drosophila* cells. Using well characterised targets (STX5, SLY1 and ROP) we have validated the system and have shown that the assay is capable of differentiating blocks in ER to Golgi and Golgi to plasma membrane transport based on proteolytic processing and accumulation of the secretory cargo in post-Golgi transport vesicles. Our experimental data suggests that there are at least three fusion complexes operating at the *Drosophila* PM (Fig 7A). The first complex consists of STX1, SNAP24/29 and Syb. The second complex consists of STX4, SNAP24/29 and Syb. The third complex consists of STX1, SNAP24 and YKT6. The reason we have excluded the possibility of a STX4, SNAP24/29, YKT6 complex is because depletion of both STX1 and Syb led to a complete block in secretion. Indicating that STX4 and YKT6 are unable to form a SNARE complex that can substitute for the loss of STX1 and Syb. Genetic interaction data also suggests that SNAP29 is unable to substitute for the loss of SNAP24 under conditions when both SNAP24 and Syb are depleted. This data suggests that the third SNARE complex specifically consists of STX1, SNAP24 and YKT6. At present it is unclear whether these SNARE complexes define parallel pathways to the plasma membrane or simply reflect the ability of these SNAREs to substitute with each other.

The most striking observation in this study is that we have uncovered an unexpected role for YKT6 in the fusion of secretory carriers with the plasma membrane. Depletion of YKT6 and Syb/VAMP3 in combination causes a complete block in secretion and leads to an accumulation of post-Golgi transport vesicles within *Drosophila* cells. YKT6 is a lipid anchored R-SNARE that has been shown to function on many pathways including ER to Golgi transport, intra-Golgi transport, endosome-vacuole fusion, endosome to Golgi transport and exosome fusion with the plasma membrane [24, 45–51]. YKT6 actively cycles on and off membranes in a palmitoylation dependant manner so potentially it is well suited to function on a wide variety of intracellular pathways [52]. Due to the promiscuous nature of YKT6 some caution must be taken when interpreting our functional data. It is possible that loss of YKT6 may be indirectly affecting post-Golgi transport and fusion at the plasma membrane. However, the simplest interpretation of our data is YKT6 is directly involved in this process as we are able to biochemically detect an interaction between YKT6 and STX1.

Using the knowledge obtained from the *Drosophila* system, we re-examined the role of R-SNAREs in constitutive secretion in mammalian cells. As previously reported, depletion of VAMP3 and other post-Golgi R-SNAREs did not perturb secretion in HeLa cells [27]. However, depletion of VAMP3 and YKT6 in combination caused a complete block in secretion. This data suggests that YKT6 and VAMP3 may be functioning in the fusion of secretory carriers with the plasma membrane in mammalian cells. We have made significant efforts to localise endogenous YKT6 and VAMP3 on post-Golgi secretory carriers. However, our attempts have been hampered by the fact the endogenus YKT6 is expressed at very low levels and over expressed YKT6 does not target correctly to membranes and remains cytoplasmic.

As expected, there is redundancy in the Q-SNAREs required for the fusion of secretory carriers with the plasma membrane. However, it is clear that certain SNAREs have a more prominent role in this process. The main Q-SNAREs at the *Drosophila* plasma membrane are STX1 and STX4 (share homology with SSO1 and 2). Depletion of STX1 causes a partial block in secretion while depletion of STX4 does not. It is unclear why STX1 is the favoured Q_a_-SNARE. It could simply be that STX1 is more abundant than STX4 or has a higher affinity for the R-SNARE on the vesicle [53]. It may also reflect the route by which the synthetic cargo traffics to the cell surface. We have also observed redundancy between the Q_bc_-SNAREs SNAP24 and SNAP29 (orthologues of Sec9). We are only able to detect a complete block in secretion when both are depleted. It has previously been shown that SNAP29 interacts with STX1. However, the complexes it forms are not SDS-resistant suggesting that they may not be fusogenic [54].

A potential problem with gene disruption and RNAi mediated depletion studies is compensation by other genes in the same family. For example, VAMP2 and 3 are upregulated in certain tissues of the VAMP8 knockout mouse and VAMP3 is upregulated in VAMP2 deficient chromafin cells isolated from VAMP2 null mice [55, 56]. Based on our immunoblotting data we did not observe any compensation between R-SNAREs when they are depleted using RNAi in *Drosophila* cells (Fig 3B). We also did not see any evidence of this in our previous work performed in HeLa cells [27]. We initially thought that STX1 and STX4 were being upregulated in STX5 and Syb depleted cells based on immunoblotting (Figs 2B and 4D). However, when the samples were directly prepared in Laemmli sample buffer, rather than a TX100 based extraction buffer, no difference in the levels of these SNAREs was observed (S2 Fig). It is possible that the change in extractability may be caused by an alteration in the localisation of the Q-SNAREs from TX100 insoluble micro-domains at the plasma membrane [57]. However, we have not tested this hypothesis. To directly assess changes in gene expression during the RNAi experiments we measured the mRNA levels several SNAREs using RT-PCR (S2 Fig). Depletion of STX1 leads to an upregulation of STX4 and Syb. However, we did not observe a significant change in the protein level of these SNAREs by immunoblotting. Thus it is unclear how significant these changes are. In the future, it will be interesting to determine how the expression levels of SNAREs, which function on the same pathway, are co-ordinated and regulated.

To validate our genetic interaction data we have interrogated a published *S*. *cerevisiae* proliferation-based genetic interaction map to determine if the yeast homologues share similar genetic interactions to those observed in *Drosophila* cells (under the assumption that constitutive secretion is essential for growth) [58]. We have detected negative genetic interactions between *Drosophila* STX1 and STX4, STX1 and Syb, Syb and SNAP24, SNAP24 and SNAP29, YKT6 and Sec22b and Syb and YKT6 (Fig 7C). Similar genetic interactions were also observed in *S*. *cerevisiae* indicating that the data generated from *Drosophila* cells is physiologically relevant and the genetic interactions are evolutionary conserved. Importantly the homologues of YKT6 and Syb/VAMP3 were also found to genetically interact in yeast (YKT6 and SNC2).

In summary, we have identified the SNARE complexes required for the fusion of constitutive secretory vesicles with the plasma membrane in *Drosophila* cells. We have uncovered a novel role for YKT6 in the fusion of secretory vesicles with the plasma membrane which is conserved from yeast to man. This observation may in part explain why RNAi and gene disruption studies in higher eukaryotes have failed to yield the expected phenotypes. In the future, it should be possible to use our secretion assay in combination with SNARE depletion as a tool to further dissect the post-Golgi pathways involved in secretion and generate post-Golgi secretory carriers for proteomic profiling.

## Materials and methods

### Antibodies

Rabbit polyclonal antibodies were raised against GFP and the cytoplasmic domains of *Drosophila* STX4, SNAP29, Syb, VAMP7 and Sec22b. The antibodies were affinity purified as in [59]. The rabbit polyclonal antibody against *Drosophila* STX7 was a generous gift from H. Krämer. The mouse monoclonal antibodies against *Drosophila* STX1 (8C3, depositors Benzer, S. and Colley, N.), ROP (4F8) and Actin (JLA20, depositor Lin, J. J.-C) were purchased from the Developmental Studies Hybridoma Bank [39]. The Rabbit polyclonal to *Drosophila* GM130 was purchased from Abcam. The mouse monoclonal to human growth hormone (2H81G10) was a generous gift from Genentech Inc.,. The rabbit polyclonal antibody that cross-reacts with *Drosophila* YKT6 was a generous gift from Jessey Hay [60]. Secondary antibodies for immunoblotting were purchased from Jackson ImmunoResearch Laboratories. Secondary antibodies for immunofluorescence microscopy were purchased from Invitrogen Molecular Probes.

### Cell culture and siRNA transfections

*Drosophila* D.mel-2 (Invitrogen) and C3 cells were maintained in Express Five® SFM media (Invitrogen,) supplemented with 100 IU/mL penicillin, 100 μg/mL streptomycin, and 2 mM glutamine (Sigma-Aldrich) at 25°C in an cooled incubator. Expression of the reporter construct in C3 cells was maintained by the addition of 5μg/mL Blasticidin (PAA Laboratories). HeLa-M and C1 cells were grown in high glucose DMEM supplemented with 10% fetal calf serum, 100 IU/mL penicillin, 100 μg/mL streptomycin, and 2 mM glutamine (Sigma-Aldrich) at 37°C in a 5% CO2 humidified incubator. Expression of the reporter construct in C1 cells was maintained by the addition of 1.66μg/mL puromycin (PAA Laboratories). siRNA transfections were performed as in Gordon et al., 2010. The sequence of the siRNA used in the experiments can be found in (S2 Table).

### Constructs and stable cell line generation

The reporter construct used to generate the C3 cell line was generated by subcloning the expression cassette from pC4S1-eGFP-FM4-FCS-hGH (Ariad Pharmaceuticals) into pAC-V5-His-A expression vector (Invitrogen). 2μg of pAC-S1-eGFP-FM4-FCS-hGH was co-transfected with 50ng of pCoBLAST into 500,000 S2 cells using the TransFast transfection reagent (Promega). A population of cells stably expressing the reporter construct was generated by the addition of 25 μg/mL blasticidin (PAA Laboratories). The cells were selected for two weeks and then autocloned into a 96 well plate using a MoFlo Flow cytometer (Beckman Coulter) based on GFP fluorescence. We were initially unsuccessful in this process until we supplemented the media with 5% FCS and put two cells in each well of the plate. 96 well plates were sealed with Parafilm M (Pechiney Plastic Packaging) to minimize evaporation during cell culture. Positive wells were identified using fluorescence microscopy. Clonal cell lines were screened for their ability to efficiently secrete the reporter construct and Clone 3 cells chosen as they have the most uniform expression of the reporter construct.

HA tagged *Drosophila* STX1 was generated using PCR and cloned into the copper inducible expression vector pMT/V5-HIS (Invitrogen). A stable population of cells was generated by co-transfecting the plasmid with pCoBLAST and selected as above.

### Amplicon synthesis and transfection

Primers for generating dsRNA amplicons were designed using the Harvard *Drosophila* RNAi Screening Center database (http://www.flyrnai.org) or the GenomeRNAi database (http://rnai2.dkfz.de/GenomeRNAi). Amplicons were chosen which were predicted to have the fewest off-target hits. Primers sequences were copied verbatim from the websites and T7 sequences added to the 5’ end of both primers for each amplicon (S2 Table). Primers were synthesized by Sigma Genosys. A cDNA library was generated from S2 cells and used as a template for amplicon synthesis. The cDNA library was made by purifying RNA from S2 cells using a QIAshredder and RNeasy Protect Mini purification kit; followed by cDNA synthesis using the QuantiTect Reverse Transcription kit (Qiagen). The DNA template for the amplicons was generated using two rounds of PCR from the cDNA library. A sample of this DNA was sequenced to confirm that the correct target had been amplified. Double stranded RNA was synthesized using the DNA template and T7 Ribomax Express RNAi System (Promega) according to manufacturers’ instructions. The reaction was cleaned up using a DNAse and RNAse digestion step followed by column purification using the RNeasy Midi kit (Qiagen). A small amount of the reaction was run on agarose gel to confirm that the amplicon was the correct size. The RNA concentration was determined using a Nanodrop spectrophotometer (Thermo Scientific). Knock downs were performed by transfecting 20μg of dsRNA into 500,000 S2 cells using TransFast (Promega). The cells were then analysed 96 hours post transfection.

### Quantitative RT-PCR

S2 cells were lysed and the RNA purified using a QIAshredder and RNeasy Protect Mini purification kit following the manufacturer’s instructions (Qiagen). The mRNA levels of specific genes were quantified using the Taqman RNA-to-C_T_ 1-Step Kit (Applied Biosystems). Pre-designed sets of primers and FAM-labeled fluorescent probes designed against target genes were ordered from Applied Biosystems, and these were used according to manufacturers’ instructions (S3 Table). qRT-PCR reactions were run on an Applied Biosystems 7900HT Fast Real-Time PCR System. To quantify knockdown efficiency, relative quantification was performed using the ΔΔC_T_ method [61]. For a list of qRT-PCR primes used in this study please see (S3 Table).

### Flow cytometry based secretion assay

Secretion of the reporter construct was induced in Clone 1 (HeLa M) or Clone 3 (S2) cells by the addition of AP21998 (Ariad Pharmaceuticals) or D/D solubilizer (Clontech). Following secretion, the cells were placed on ice for 10 minutes to halt vesicle trafficking. C1 cells were detached using cold EDTA-Trypsin solution (PAA Laboratories) for 2 hours on ice. C3 cells are semi-adherent so were detached with pipetting. The fluorescence of the cells was measured using a BD FacsCalibur equipped with an HTS 96-well sampling robot (BD Biosciences). Live cells were gated using forward and side scatter and dead cell exclusion (2 μg/mL 7-AAD for clone 1 cells or 1 μg/mL PI for clone 3 cells) (Molecular Probes Invitrogen). A minimum of 2000 cells were analysed for each sample. FlowJo (Treestar) was used to calculate the geometric mean fluorescence for each sample. GraphPad Prizm (GraphPad Software) was used for generating statistics and graphs. Each sample is set up in duplicate. One sample receives AP21998 or D/D solubilizer and the other does not. The percentage of cargo remaining after secretion is then calculated by taking a ratio between the two samples.

### Immunoblotting

To measure secretion by immunoblotting, equal numbers of C3 cells were resuspended in fresh media containing AP21998 and incubated for 80 minutes at 25°C. Secretion was halted by cooling the cells to 4°C and the media and cells collected by centrifugation. The media and cells were solubilized in Laemmli sample buffer and separated using SDS-PAGE. Proteins were transferred overnight onto PVDF membranes using wet transfer conditions. The membranes were blocked using 5% milk, 1% Tween-20 in PBS and probed with antibodies against actin (loading control) and growth hormone. Secondary antibodies conjugated to HRP were used to detect the primary antibodies and Supersignal West Pico Substrate (Pierce) used to develop the blots. Super RX Medical X-Ray Film (Fujifilm) was used to capture the signal and densitometry performed using ImageJ software. To evaluate knock down efficiency, C3 cells were counted and equal numbers of cells collected by centrifugation. The cells were resuspended in TX100-based extraction buffer (100 mM NaCl, 5 mM MgCl_2_, 50 mM Tris pH 7.4, 1% TX100), incubated for 15 minutes on ice, centrifuged at 15,000 g for 15 min at 4°C. The supernatants were normalised for protein concentration using the Bradford protein assay, (BIO-RAD), boiled in reducing SDS sample buffer and separated by polyacrylamide gel electrophoresis. Antibodies against actin (loading control) and SNAREs were used to probe the membranes.

### Immuoprecipitations and mass spectrometry

To isolate HA-tagged syntaxin 1/SNARE complexes, cells were resuspended in lysis buffer (100 mM NaCl, 5 mM MgCl_2_, 50 mM Tris pH 7.4, 0.5% Igepal CA-630) with a complete protease inhibitor tablet (Roche) and incubated for 30 minutes. Insoluble material was removed by centrifugation at 5,000 rpm for 5 minutes and then followed by centrifugation at 50,000 rpm for 30 minutes. The lysate was then passed through a 0.2 μm syringe filter. Cleared lysate was incubated for two hours with anti-HA resin (Roche). Following multiple wash steps, samples were eluted twice with one column volume of 1 mg/mL HA-peptide (Roche) and acetone precipitated. The samples were then solubilized in Laemmli sample buffer and separated using SDS-PAGE. The gel was stained using SYPRO Ruby (Molecular Probes Invitrogen) and the bands visulaised using a Typhoon Trio Variable Mode Imager (GE Healthcare). The bands were excised using a scalpel blade and in-gel trypsin digestion performed. Analysis was performed using an AB Sciex 4800 MALDI TOF/TOF. The instrument is configured to acquire an MS spectrum between m/z 700 and 4000. From these MS spectra 7 peptides above a predetermined s/n threshold are selected for fragmentation. The MS spectra of intact peptides are used to determine protein identity by peptide mass fingerprinting (PMF) using the MASCOT search engine (NCBInr database 20/10/2010, 12061831 sequences). For further confirmation, the MSMS spectra are used to perform fragment ion searches to determine peptide sequence but if they fail to yield any identifications, it may be because peptides above the s/n threshold gave poor fragmentation patterns.

### Fluorescence microscopy

C3 cells were grown on 13 mm No. 1 round coverslips (VWR) coated with Concanavalin-A (Sigma-Aldrich) and allowed to adhere over night. Cells were incubated in the presence of AP21998 or D/D solubilizer for 80 minutes. Cells were then fixed and stained as described in [62]. Coverslips were mounted using ProLong Gold (Molecular Probes Invitrogen) and sealed with clear nail polish. Images were captured using either a 63x or 100x oil objective on a Zeiss Axioplan fluorescence microscope (Zeiss) equipped with a Hamamatsu Orca-R2 C10600 camera (Hamamatsu Photonics), and SEDAT quad pass filter set (Chroma). The brightness and contrast of microscopy images were adjusted using ImageJ (NIH).

## Supporting information

S1 Fig STX5 and SLH are efficiently depleted using RNAi and lead to a block in ER to Golgi transport. A) Clone 3 cells were mock transfected (TransFast only) or transfected with dsRNA targeting STX5 and SLH. After 96 hours, the cells were harvested and the mRNA levels of STX5 and SLH determined using qRT-PCR. Error bars indicate the experimental range between duplicate experiments. B) Clone 3 cells were grown on coverslips and either mock transfected or transfected with dsRNA targeting STX5. After 96 hours, the cells were incubated with AP21998 at 25°C for 80 minutes. The cells were then fixed and stained for the Golgi marker GM130. There is some heterogeneity in the expression level of the reporter construct between cells. In cells with low expression levels the secretory reporter appears reticular and in cells with higher levels the ER appears more distended and vesicular in nature (STX5-GFP image).
(PDF)

S2 Fig Representative histograms, immunoblots and RT-PCR results for STX1, STX4 and Syb RNAi experiments. A) Clone 3 cells were mock transfected (TransFast only) or transfected with dsRNA targeting the indicated genes. After 96 hours, the cells were incubated with AP21998 at 25°C for 80 minutes and their mean fluorescence determined using flow cytometry. The red histogram indicates the fluorescent intensity of the control sample, no AP21998 and the blue histogram shows the fluorescent intensity of the cells incubated with AP21998. B) Clone 3 cells were mock transfected (TransFast only) or transfected with dsRNA targeting the indicated genes. After 96 hours, the cells were directly solubilised in Laemmli sample buffer and the protein concentration normalised using an actin loading control. C) Clone 3 cells were mock transfected (TransFast only) or transfected with dsRNA targeting STX1, STX4 and Syb. After 96 hours, the cells were harvested and the mRNA levels of STX4 and Syb determined using qRT-PCR. Error bars indicate the SD of two biological repeats.
(PDF)

S3 Fig Representative histograms for R-SNARE RNAi experiments. Clone 3 cells were mock transfected (TransFast only) or transfected with dsRNA targeting the indicated genes. After 96 hours, the cells were incubated with AP21998 at 25°C for 80 minutes and their mean fluorescence determined using flow cytometry. The red histogram indicates the fluorescent intensity of the control sample, no AP21998 and the blue histogram shows the fluorescent intensity of the cells incubated with AP21998.
(PDF)

S4 Fig Representative histograms for YKT6 and Sec22b RNAi experiments. Clone 3 cells were mock transfected (TransFast only) or transfected with dsRNA targeting the indicated genes. After 96 hours, the cells were incubated with AP21998 at 25°C for 80 minutes and their mean fluorescence determined using flow cytometry. The red histogram indicates the fluorescent intensity of the control sample, no AP21998 and the blue histogram shows the fluorescent intensity of the cells incubated with AP21998.
(PDF)

S5 Fig Representative histograms for SNAP RNAi experiments. Clone 3 cells were mock transfected (TransFast only) or transfected with dsRNA targeting the indicated genes. After 96 hours, the cells were incubated with DD solubiliser at 25°C for 80 minutes and their mean fluorescence determined using flow cytometry. The red histogram indicates the fluorescent intensity of the control sample, no DD solubiliser and the blue histogram shows the fluorescent intensity of the cells incubated with DD solubiliser.
(PDF)

S1 Table Summary of alternate amplicon data. (DOCX)

S2 Table Amplicon primer sequences. (DOCX)

S3 Table qRT-PCR Primers. (DOCX)
