# Supplementary figures and images for: VAMP3/Syb and YKT6 are required for the fusion of constitutive secretory carriers with the plasma membrane
Source: PLoS Genet. 2017 Apr 12;13(4):e1006698. doi: 10.1371/journal.pgen.1006698 (PMC5406017; doi:10.1371/journal.pgen.1006698)

S1 Fig

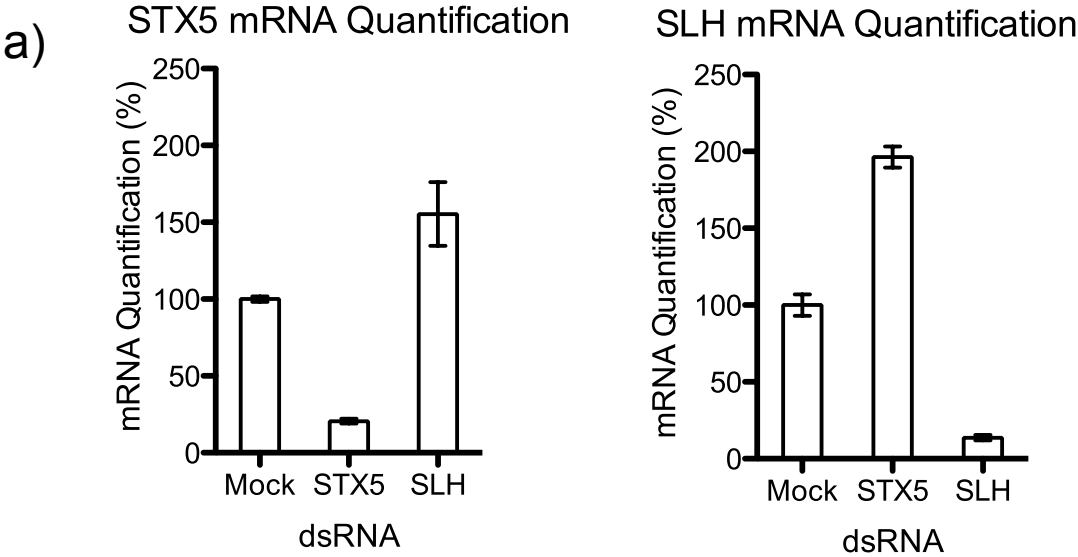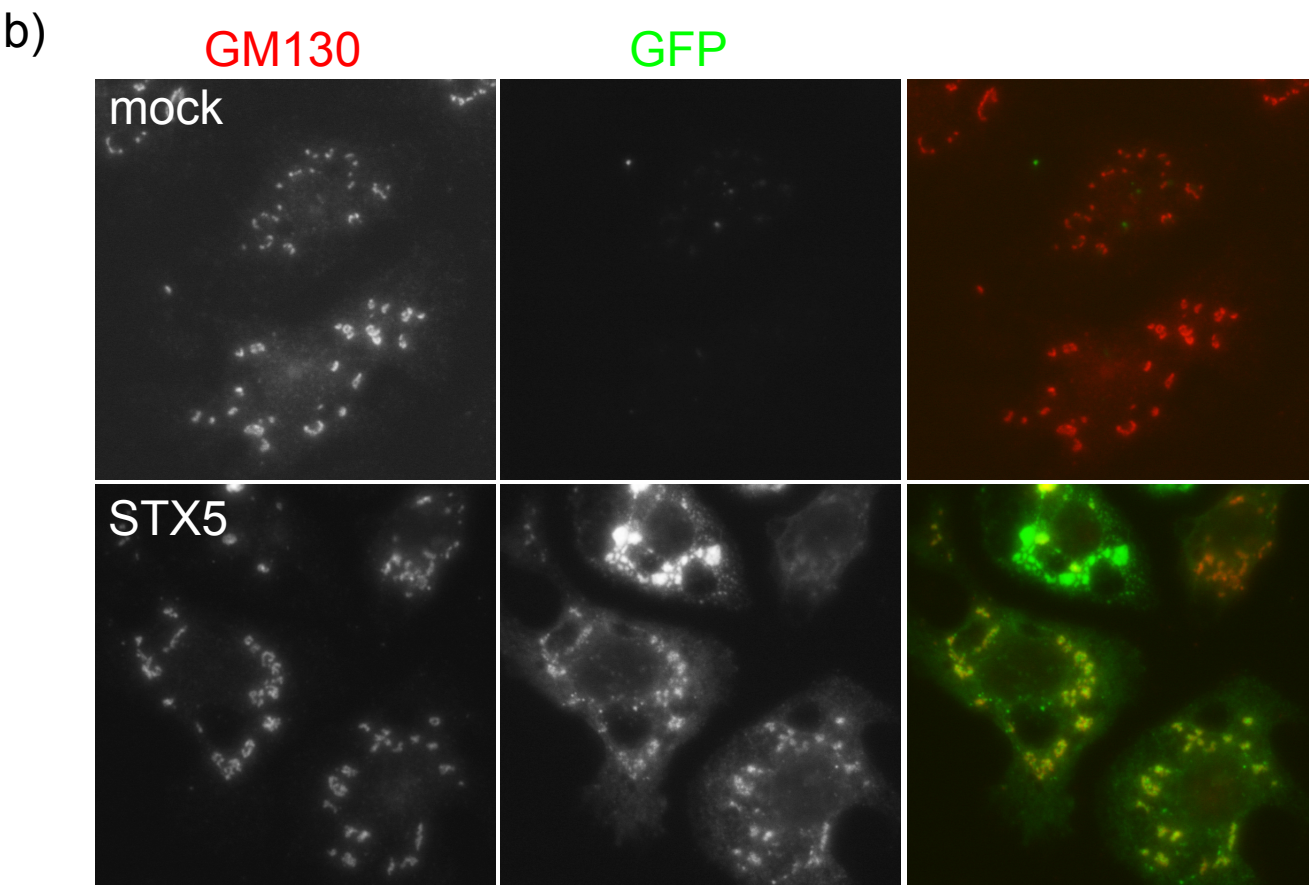

Supplement: S1 Fig — A) Clone 3 cells were mock transfected (TransFast only) or transfected with dsRNA targeting STX5 and SLH. After 96 hours, the cells were harvested and the mRNA levels of STX5 and SLH determined using qRT-PCR. Error bars indicate the experimental range between duplicate experiments. B) Clone 3 cells were grown on coverslips and either mock transfected or transfected with dsRNA targeting STX5. After 96 hours, the cells were incubated with AP21998 at 25°C for 80 minutes. The cells were then fixed and stained for the Golgi marker GM130. There is some heterogeneity in the expression level of the reporter construct between cells. In cells with low expression levels the secretory reporter appears reticular and in cells with higher levels the ER appears more distended and vesicular in nature (STX5-GFP image). (PDF) [file pgen.1006698.s001.pdf]

# S2 Fig

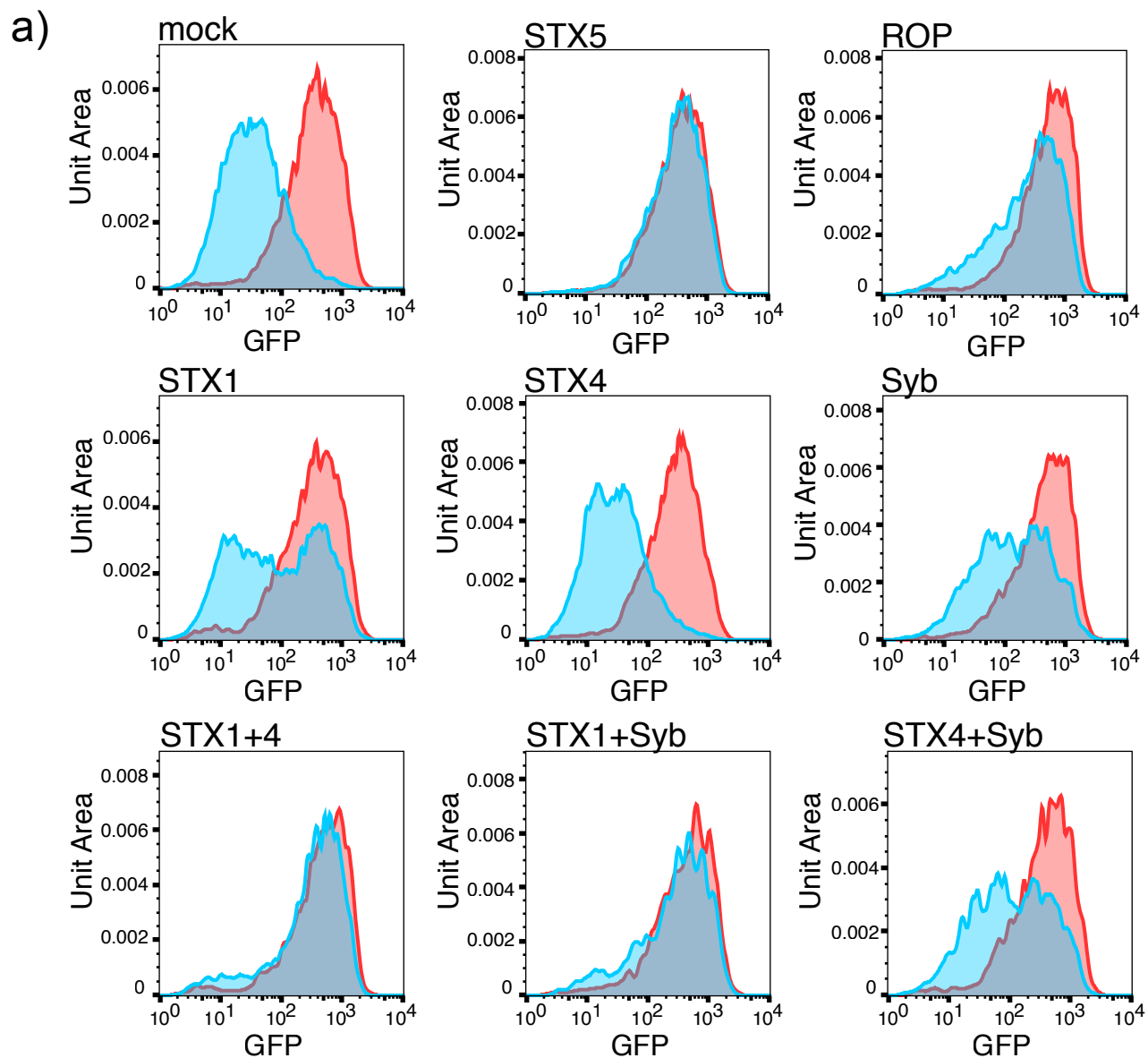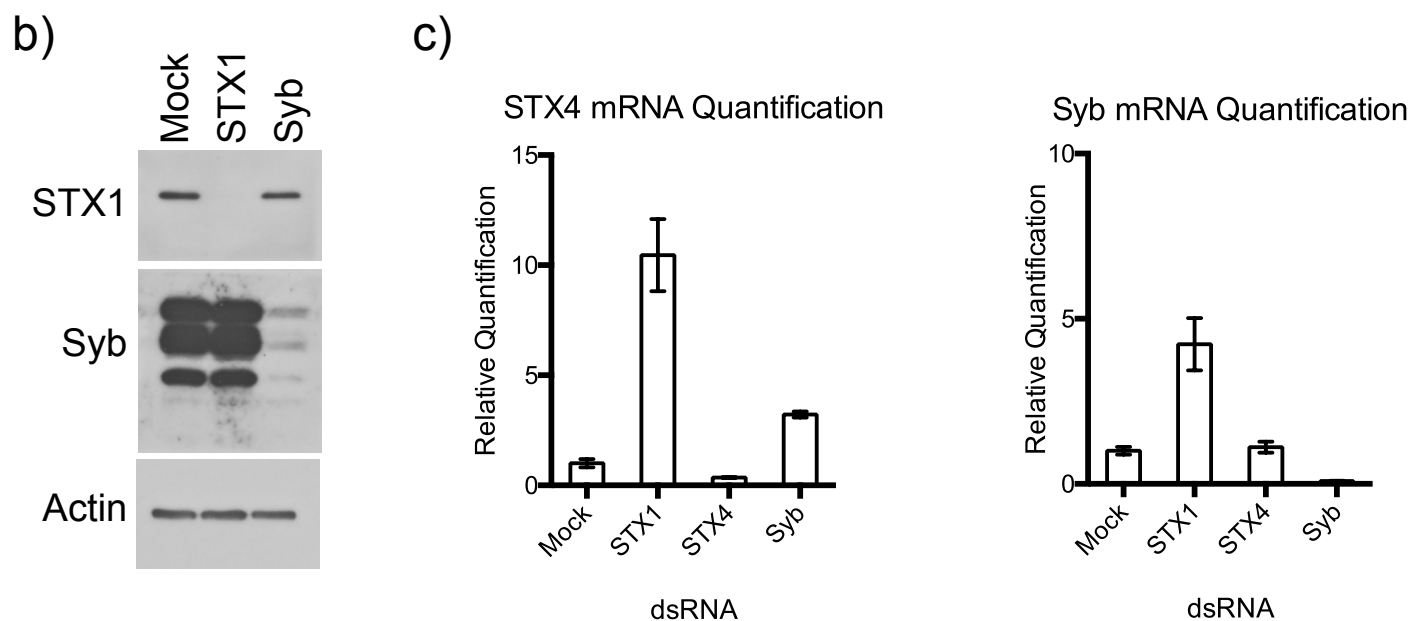

Supplement: S2 Fig — A) Clone 3 cells were mock transfected (TransFast only) or transfected with dsRNA targeting the indicated genes. After 96 hours, the cells were incubated with AP21998 at 25°C for 80 minutes and their mean fluorescence determined using flow cytometry. The red histogram indicates the fluorescent intensity of the control sample, no AP21998 and the blue histogram shows the fluorescent intensity of the cells incubated with AP21998. B) Clone 3 cells were mock transfected (TransFast only) or transfected with dsRNA targeting the indicated genes. After 96 hours, the cells were directly solubilised in Laemmli sample buffer and the protein concentration normalised using an actin loading control. C) Clone 3 cells were mock transfected (TransFast only) or transfected with dsRNA targeting STX1, STX4 and Syb. After 96 hours, the cells were harvested and the mRNA levels of STX4 and Syb determined using qRT-PCR. Error bars indicate the SD of two biological repeats. (PDF) [file pgen.1006698.s002.pdf]

# S3 Fig

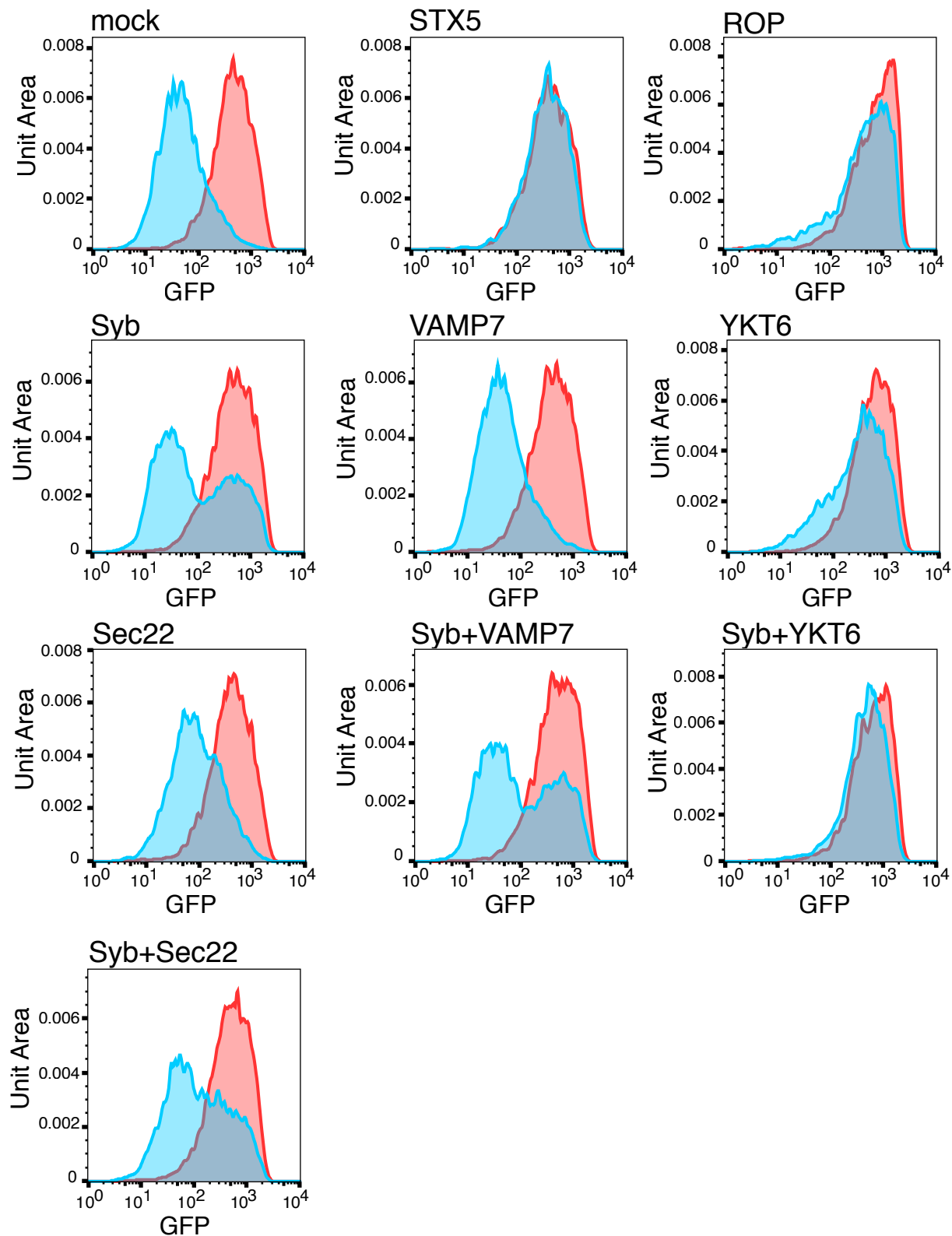

Supplement: S3 Fig — Clone 3 cells were mock transfected (TransFast only) or transfected with dsRNA targeting the indicated genes. After 96 hours, the cells were incubated with AP21998 at 25°C for 80 minutes and their mean fluorescence determined using flow cytometry. The red histogram indicates the fluorescent intensity of the control sample, no AP21998 and the blue histogram shows the fluorescent intensity of the cells incubated with AP21998. (PDF) [file pgen.1006698.s003.pdf]

# S4 Fig

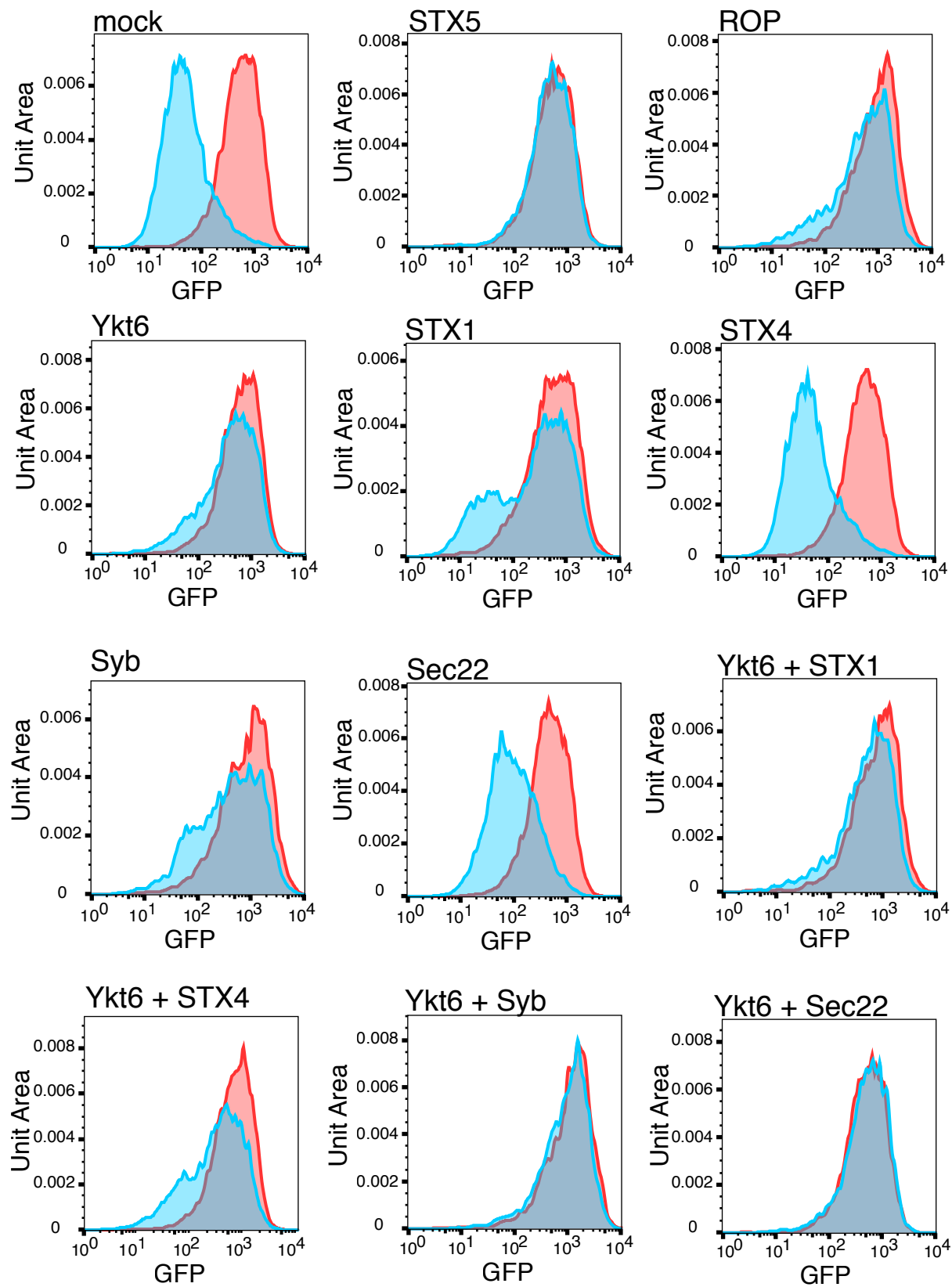

Supplement: S4 Fig — Clone 3 cells were mock transfected (TransFast only) or transfected with dsRNA targeting the indicated genes. After 96 hours, the cells were incubated with AP21998 at 25°C for 80 minutes and their mean fluorescence determined using flow cytometry. The red histogram indicates the fluorescent intensity of the control sample, no AP21998 and the blue histogram shows the fluorescent intensity of the cells incubated with AP21998. (PDF) [file pgen.1006698.s004.pdf]

S5 Fig

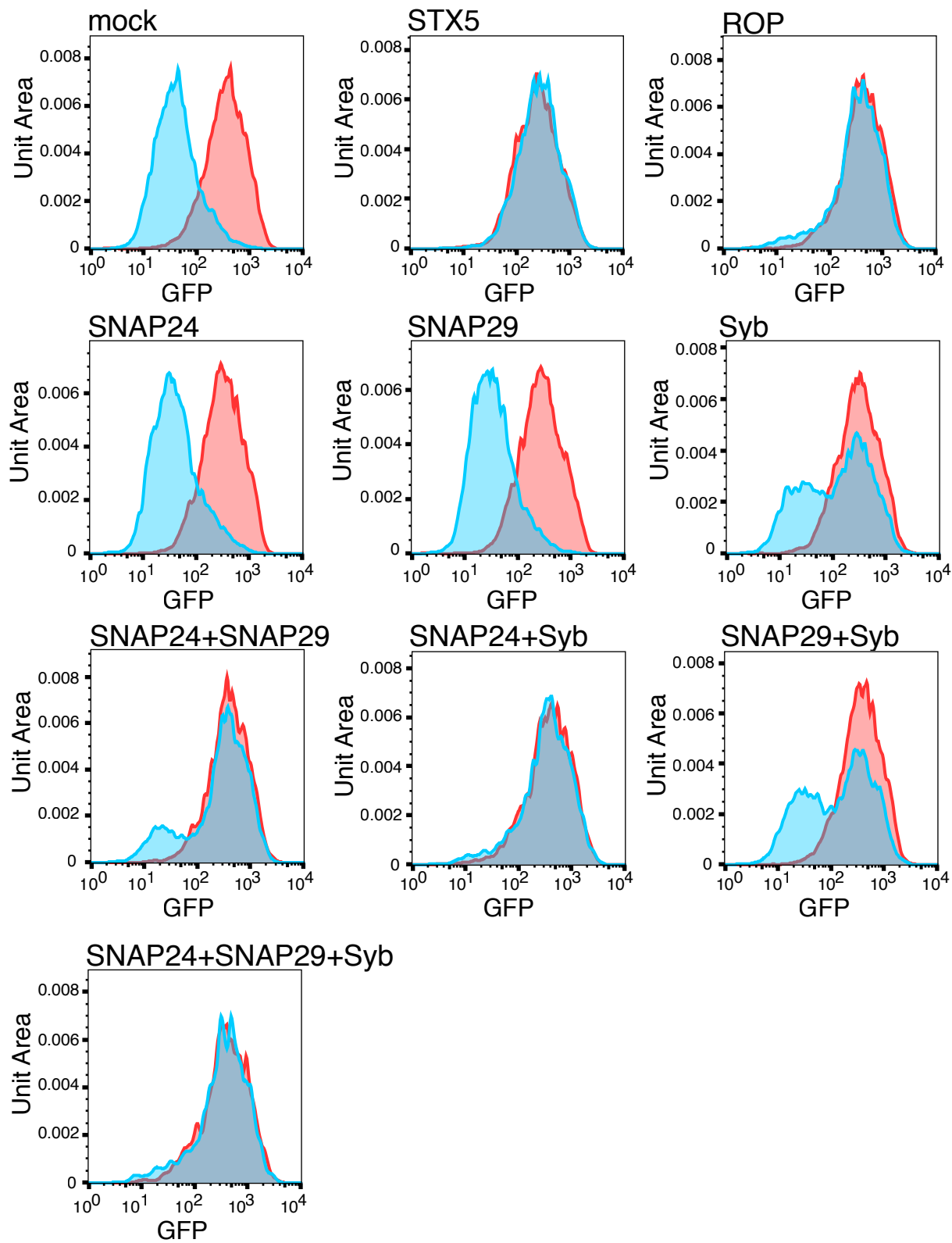

Supplement: S5 Fig — Clone 3 cells were mock transfected (TransFast only) or transfected with dsRNA targeting the indicated genes. After 96 hours, the cells were incubated with DD solubiliser at 25°C for 80 minutes and their mean fluorescence determined using flow cytometry. The red histogram indicates the fluorescent intensity of the control sample, no DD solubiliser and the blue histogram shows the fluorescent intensity of the cells incubated with DD solubiliser. (PDF) [file pgen.1006698.s005.pdf]
