# Supplementary material for: VAMP3/Syb and YKT6 are required for the fusion of constitutive secretory carriers with the plasma membrane
Source: PLoS Genet. 2017 Apr 12;13(4):e1006698. doi: 10.1371/journal.pgen.1006698 (PMC5406017; doi:10.1371/journal.pgen.1006698)
Supplement: S1 Table — (DOCX) [file pgen.1006698.s006.docx]

**S1 Table. Summary of alternate dsRNA amplicon data**

| **Gene Target** | **Amplicon 1**  **(Used for data in main text)** | **Amplicon 2** | **Amplicon 3** |
| --- | --- | --- | --- |
| STX5 | Blocks secretion | Partially blocks secretion |  |
| SLY1 | Blocks secretion | Partially blocks secretion |  |
| ROP | Blocks secretion | Partially blocks secretion |  |
| STX1 | Blocks secretion in combination with STX4-1 and Syb-1 | Not a complete knock down, determined by western blotting | Not a complete knock down, determined by western blotting |
| STX4 | Blocks secretion in combination with STX1-1 | Not tested |  |
| Syb | Blocks secretion in combination with STX1-1, YKT6-1, and SNAP24-1 | Blocks secretion in combination with STX1-1, YKT6-1, and YKT6-2 |  |
| YKT6 | Blocks secretion in combination with Syb-1, Syb-2, and Sec22-1 | Blocks secretion in combination with Syb-2 |  |
| VAMP7 | No block.  Knock down confirmed by western blotting | No block. |  |
| Sec22b | Blocks secretion in combination with YKT6-1 | Not tested |  |
| SNAP24 | Blocks secretion in combination with SNAP29-1 and Syb-1 | Partial block in secretion in combination with SNAP29-2 and Syb-1 |  |
| SNAP29 | Blocks secretion in combination with SNAP24-1 | Partial block in secretion in combination with SNAP24-2 |  |

As determined by flow cytometry.
