# Supplementary material for: VAMP3/Syb and YKT6 are required for the fusion of constitutive secretory carriers with the plasma membrane
Source: PLoS Genet. 2017 Apr 12;13(4):e1006698. doi: 10.1371/journal.pgen.1006698 (PMC5406017; doi:10.1371/journal.pgen.1006698)
Supplement: S2 Table — (DOCX) [file pgen.1006698.s007.docx]

**S2 Table**

**Amplicon primer sequences: dsRNA synthesis for Drosophila gene knockdowns**

| Amplicon | Length (bp) | Primer “R”  (with T7 sequence) | Primer “S”  (with T7 sequence) | Amplicon Designation  (DRSC or Genome RNAi) | Predicted Off Target Knockdowns |
| --- | --- | --- | --- | --- | --- |
| **SM Proteins** |  |  |  |  |  |
| ROP | 306 | GCTTCTAATACGACTCACTATAGATCGCTATGCCCATGTCTTC | GCTTCTAATACGACTCACTATAGAATCGATGTTCCTGTCCCAG | DRSC26024 | 0 |
| ROP-2 | 207 | GCTTCTAATACGACTCACTATAGAGGACATCATGGAGGATTGC | GCTTCTAATACGACTCACTATAGACCGCATCTCGGACATACTC | DRSC31662 | 0 |
| SLH | 507 | GCTTCTAATACGACTCACTATAGAAAACCCAAGGCCTGTGAC | GCTTCTAATACGACTCACTATAGGCCTGCAGCGCCTCC | DRSC00789 | 0 |
| SLH-2 | 504 | GCTTCTAATACGACTCACTATAGGGATTCAGCAGGACTTCTCG | GCTTCTAATACGACTCACTATAGAATCCATGTTCCTGTCCAGC | DRSC33353 | 0 |
| **Qa-SNAREs** |  |  |  |  |  |
| STX1 | 513 | GCTTCTAATACGACTCACTATAGACTCGGCCATCCTGTCC | GCTTCTAATACGACTCACTATAGTCTCGCCCTGCGACTC | DRSC15359 | 0 |
| STX1-2 | 518 | GCTTCTAATACGACTCACTATAGGTCTCTCGCAAAAGTGGAA | GCTTCTAATACGACTCACTATAGTGCACCTTGTCGATCATCC | DRSC21554 | 0 |
| STX1-3 | 198 | GCTTCTAATACGACTCACTATAGGGGAAAATCGCGAAACAATA | GCTTCTAATACGACTCACTATAGCGATTCAGGGTGTGTGTTTG | DRSC31677 | 0 |
| STX4 | 301 | GCTTCTAATACGACTCACTATAGAACTTGGAGACGATGAACCG | GCTTCTAATACGACTCACTATAGCGTTTTTGTGCCACAGATTG | DRSC27586 | 0 |
| STX5 | 507 | GCTTCTAATACGACTCACTATAGAAAAAGAAGAGCTTATTTGATGA | GCTTCTAATACGACTCACTATAGCTCAACGATGGTAGATTCTATAT | DRSC03432 | 0 |
| STX5-2 | 404 | GCTTCTAATACGACTCACTATAGATAATTACGTCCAGCAGCGG | GCTTCTAATACGACTCACTATAGTTTACAATCGTTTCGGGAGG | DRSC40015 | 0 |
| STX7 | 232 | GCTTCTAATACGACTCACTATAGAAGAATTAGAGCCTTCCGGC | GCTTCTAATACGACTCACTATAGGCCGGTGAATACTCCTCAAA | DRSC31679 | 0 |
| **R-SNAREs** |  |  |  |  |  |
| Sec22b | 362 | GCTTCTAATACGACTCACTATAGGTACAAGAAGGACGCCAAGC | GCTTCTAATACGACTCACTATAGTCCCAGACCGTAAATAACGC | DRSC39605 | 0 |
| Syb | 248 | GCTTCTAATACGACTCACTATAGACAATGCAGCCCAGAAGAA | GCTTCTAATACGACTCACTATAGGAGCAGCACAACGGCTAT | DRSC07559 | 1  (VHA100-3, not expressed in S2 cells) |
| Syb-2 | 234 | GCTTCTAATACGACTCACTATAGCTAGTAGTGACAGCGGCAGTGG | GCTTCTAATACGACTCACTATAGCATTCTGGTTAAATATATGCTAGACGC | OBS, 67602 | 0 |
| VAMP7 | 197 | GCTTCTAATACGACTCACTATAGGCGGGCCTTCCTCTTCT | GCTTCTAATACGACTCACTATAGTGTCTTTAAGCTCGTCAATCT | DRSC06599 | 0 |
| VAMP7-2 | 292 | GCTTCTAATACGACTCACTATAGCGGCAAAAGCAATTGAAAAT | GCTTCTAATACGACTCACTATAGTTTGCTTATCGGGGAATGAG | DRSC40244 | 0 |
| Ykt6 | 591 | GCTTCTAATACGACTCACTATAGCGAGGCGCGTCTCCT | GCTTCTAATACGACTCACTATAGGCTCTGCAGCGACAATTT | DRSC17970 | 0 |
| Ykt6-2 | 586 | GCTTCTAATACGACTCACTATAGGTGTCATGTCTATGTGCGGG | GCTTCTAATACGACTCACTATAGAATCATCAAAGCCCAAGTCG | DRSC26017 | 0 |
| **Qbc-SNAREs** |  |  |  |  |  |
| SNAP24 | 500 | GCTTCTAATACGACTCACTATAGCAGGTGGCCGATGAATCC | GCTTCTAATACGACTCACTATAGATGCGATCAACCTGTTTGTT | DRSC16864 | 0 |
| SNAP24-2 | 200 | GCTTCTAATACGACTCACTATAGTTAAATATGCACAACGGCCA | GCTTCTAATACGACTCACTATAGGGTTTGTTACGCTTGCGTTT | DRSC36023 | 0 |
| SNAP29 | 498 | GCTTCTAATACGACTCACTATAGTCTACGAAACCCAGGAGGTG | GCTTCTAATACGACTCACTATAGGCTGCACATCTCCTCCAGAT | DRSC24658 | 0 |
| SNAP29-2 | 204 | GCTTCTAATACGACTCACTATAGCAGTGCACGATCACTTCGAT | GCTTCTAATACGACTCACTATAGCCCTTCGCTTCTCAGCATAG | DRSC34865 | 0 |

**siRNA sequences targeting human SNAREs**

| **Gene Targeted** | **Catalog Number** | **Sequence** |
| --- | --- | --- |
| VAMP3 | J-011934-08 (Dharmacon) | UCAAGUAGAUGAGGUGGUG |
| VAMP4 | J-004241-06 (Dharmacon) | GGACAAAUCAGAAAGCUUA |
| VAMP7 | J-020864-06 (Dharmacon) | GAACGUUCCCGAGCCUUUA |
| VAMP8 | J-013503-06 (Dharmacon) | GUCCUUAUCUGCGUGAUUG |
| YKT6 | J-019237-10 (Dharmacon) | CUAUAAAACUGCCCGGAAA |
| SEC22 | J-011911-11 (Dharmacon) | GGUUGUGACUUUAGUAUAC |
| STX5 | STX5A_3 (Ambion) | GGAACAGGAGGAAACCAUU |
