# Supplementary material for: VAMP3/Syb and YKT6 are required for the fusion of constitutive secretory carriers with the plasma membrane
Source: PLoS Genet. 2017 Apr 12;13(4):e1006698. doi: 10.1371/journal.pgen.1006698 (PMC5406017; doi:10.1371/journal.pgen.1006698)
Supplement: S3 Table — (DOCX) [file pgen.1006698.s008.docx]

**S3 Table. qRT-PCR Primers**

| **Gene** | **Assay ID** | **Label** | **Sequence of Fluorescent Probe** | **Length** |
| --- | --- | --- | --- | --- |
| Rpl32 | Dm02151827_g1 | FAM | CTAAGCTGTCGCACAAATGGCGCAA | 72 |
| SLH | Dm01808182_g1 | FAM | ACGCTGCATGTGCAACTGCACTCGG | 73 |
| Syntaxin 5 | Dm01817827_g1 | FAM | TCACAATGTTGGCCAAAAAGAAGAG | 67 |
| Syb | Dm01818113_m1 | FAM | TCATCATCGTTCTGGTGTCCGTTTG | 62 |
| STX4 | Dm01843690_g1 | FAM | AGAAGAAGGCGCGCAAGAAAAAGAT | 60 |
